# Identification by proximity labeling of novel lipidic and proteinaceous potential partners of the dopamine transporter

**DOI:** 10.1007/s00018-021-03998-1

**Published:** 2021-10-28

**Authors:** Dolores Piniella, Elena Martínez-Blanco, David Bartolomé-Martín, Ana B. Sanz-Martos, Francisco Zafra

**Affiliations:** 1 Present Address: Centro de Biología Molecular Severo Ochoa and Departamento de Biología Molecular, Facultad de Ciencias, Consejo Superior de Investigaciones Científicas and Universidad Autónoma de Madrid, C / Nicolás Cabrera 1, 28049 Madrid, Spain; 2 IdiPAZ, Instituto de Salud Carlos III, Madrid, Spain; 3 Departamento de Ciencias Farmacéuticas y de la Salud, Facultad de Farmacia, Universidad CEU-San Pablo, CEU Universities, 28925 Madrid, Spain; 4 Present Address: Departamento de Bioquímica, Microbiología, Biología Celular y Genética, Universidad de La Laguna, Tenerife, Spain

**Keywords:** Dopamine transporters, Proteomics, Intracellular trafficking, Phosphoinositides

## Abstract

Dopamine (DA) transporters (DATs) are regulated by trafficking and modulatory processes that probably rely on stable and transient interactions with neighboring proteins and lipids. Using proximity-dependent biotin identification (BioID), we found novel potential partners for DAT, including several membrane proteins, such as the transmembrane chaperone 4F2hc, the proteolipid M6a and a potential membrane receptor for progesterone (PGRMC2). We also detected two cytoplasmic proteins: a component of the Cullin1-dependent ubiquitination machinery termed F-box/LRR-repeat protein 2 (FBXL2), and the enzyme inositol 5-phosphatase 2 (SHIP2). Immunoprecipitation (IP) and immunofluorescence studies confirmed either a physical association or a close spatial proximity between these proteins and DAT. M6a, SHIP2 and the Cullin1 system were shown to increase DAT activity in coexpression experiments, suggesting a functional role for their association. Deeper analysis revealed that M6a, which is enriched in neuronal protrusions (filopodia or dendritic spines), colocalized with DAT in these structures. In addition, the product of SHIP2 enzymatic activity (phosphatidylinositol 3,4-bisphosphate [PI(3,4)P_2_]) was tightly associated with DAT, as shown by co-IP and by colocalization of mCherry-DAT with a specific biosensor for this phospholipid. PI(3,4)P_2_ strongly stimulated transport activity in electrophysiological recordings, and conversely, inhibition of SHIP2 reduced DA uptake in several experimental systems including striatal synaptosomes and the dopaminergic cell line SH-SY5Y. In summary, here we report several potential new partners for DAT and a novel regulatory lipid, which may represent new pharmacological targets for DAT, a pivotal protein in dopaminergic function of the brain.

**Supplementary Information:**

The online version contains supplementary material available at 10.1007/s00018-021-03998-1.

## Introduction

The dopamine (DA) transporter (DAT) is a neuronal protein that controls DA flux in brain, and is, therefore, a major regulator of dopaminergic functions including control of movement, cognition, mood or reward. Mutations in the coding and non-coding regions of the DAT gene have been associated with various motor and neuropsychiatric disorders [1, 2]. Moreover, therapeutic or abuse drugs affect DAT activity either by inhibition (in the case of Ritalin or cocaine) or by promoting heteroexchange and the consequent release of DA into the synaptic cleft (in the case of amphetamines). Both mechanisms result in potentiation of the activity of diverse DA receptors during drug therapy or abuse [3, 4]. A member of the SLC6 family of neurotransporters, DAT co-transports one molecule of DA with two sodium ions and one chloride. Recent advances in structural biology have revealed the 3-D structure of DAT, improving our understanding not only of the mechanism of substrate translocation but also those of interacting drugs. The 3-D structure of Drosophila DAT confirmed the bioinformatics prediction that DAT would fold much like the prokaryotic orthologue of SLC6 transporters, LeuT, described more than 15 years ago [5, 6].

DAT is mainly expressed in dopaminergic neurons that project from the *substantia nigra* into the striatum. The protein is asymmetrically distributed in these neurons, with a higher concentration in axon terminals, although significant levels are also found in dendrites and dendritic spines in the *substantia nigra* [7]. Biochemical and cell biology studies performed over the past three decades have revealed DAT’s rich and dynamic interactome, which regulates its transport activity and intracellular trafficking to and from the surface of neurons as well as its lateral mobility between microdomains of the plasma membrane. DAT trafficking and activity is also regulated by signaling pathways initiated by the activation of surface receptors, such as G protein-coupled membrane receptors (including DA D2 receptors, mGluR5 or muscarinic M5 receptors, or the βγ G-protein subunits) or tyrosine kinase receptors (including insulin-like growth factor receptor [IGFR]-1 and the RET receptor) [8–15]. These signaling pathways involve enzymes, such as kinases (e.g., PKC, CaMKII, AKT or MAPK), phosphatases (e.g., PP2A), ubiquitin ligases (e.g., Nedd4-2), palmitoylating enzymes or nitric oxide-releasing enzymes, among other [10, 16–24]. Although the mechanisms that translate the signals of these enzymes into an impact on DAT dynamics are poorly characterized, several proteins have been identified that participate in trafficking events, including synaptic and non-synaptic proteins, such as syntaxin1A [25], α-Synuclein [26, 27], Hic-5, PICK1, SCAMP, Flotillin-1, Snapin or Ctr9 [28–33]. In addition, the secretory pathway that delivers DAT to the cell surface is controlled by the chaperone Hsc70, the ubiquitin ligase Parkin, or the coatomer component Sec24D [34–36]. These interactors ensure that DAT is correctly folded and oligomerized, both essential processes, since misfolded transporters get stuck in the ER and may promote infantile/juvenile parkinsonism [37]. Apart from these proteinaceous influences, DAT is also regulated by interactions with the lipidic environment. Thus, by interacting with specific charged residues of DAT (Lys 3, 5 and 243), the phospholipid PI(4,5)P_2_ (phosphatidylinositol 4,5-bisphosphate) potentiates the efflux of DA promoted by amphetamines [17, 38]. In addition, membrane cholesterol is a critical molecule for the function of DAT, as binding of cholesterol to defined pockets in the transmembrane region of the transporter stabilizes its outward-facing conformation [39–42]. Recent super-resolution microscopy observations revealed that DAT is dynamically sequestered within cholesterol-dependent nanodomains at the plasma membrane of presynaptic varicosities and neuronal projections of dopaminergic neurons, and this localization depends on the activity of ionotropic glutamate receptors [43]. It has also been suggested that interactions with axonal cholesterol could be the cause of the immobilization of DAT in axons, a location reached mainly by lateral diffusion from the somatodendritic compartment [44]. The mutation R615C, which results in a shift of DAT to areas devoid of GM1 ganglioside and flotillin, impacts transporter regulation and is a risk factor for the development of attention deficit/hyperactivity disorder [45, 46], an example of the importance of the interactions of DAT with the different lipid microdomains of the membrane. Thus, these data support that the proteins and mechanisms in charge of maintaining lipid homeostasis in the plasma membrane may regulate DAT by controlling its kinetics and localization. Furthermore, many of these regulatory interactions are affected by substrates and inhibitors, and may become unbalanced under pathological and drug abuse conditions. Understandably, elucidation of these regulatory mechanisms has been the goal of many laboratories for decades.

Despite the number of already reported partners, and considering DAT’s complex behavior, the list is expected to growth as new techniques for the study of protein–protein interactions become available, especially to include those proteins with weak or transient interactions that might have been overlooked by classical proteomics. In this sense, during the last few years, researchers have developed several techniques based on the principle of proximity labelling. One of these techniques, proximity-dependent biotin identification (BioID), is based on the ability of a promiscuous bacterial biotin ligase (BirA R118G, or BirA*) to biotinylate proteins located either transiently or permanently in the neighborhood of the bait protein [47, 48]. Indeed, we recently used BioID to uncover new potential partners of the glutamate transporter GLT-1 [49] and to reveal that the potassium channel Kv7.2/3 is associated with both GLT-1 and DAT [50]. In the current study, we describe new potential partners of DAT uncovered by BioID, having found interactions with potential physiological importance that include cytoplasmic and membrane proteins as well as a membrane phospholipid. Although additional studies are necessary to evaluate the functional significance of some of the discovered interactions in animal models, they contribute to a better understanding of DAT function and open up potential new pharmacological targets.

## Materials and methods

### Reagents

[^3^H]-DA was purchased from Perkin Elmer and the protein standards for sodium dodecyl sulfate–polyacrylamide gel electrophoresis (SDS–PAGE, Precision Plus Standards), nitrocellulose sheets, Bradford protein assays and Clarity™ ECL Western Blotting substrate were all obtained from Bio-Rad. and phenylmethanesulfonyl fluoride (PMSF), bacterial protease inhibitor cocktail, the Expand High Fidelity PCR system and all necessary restriction enzymes were obtained from Roche Applied Science. Plasmids pCDNA3 and pCDNA3.1/MycHis-A, fetal calf serum (FCS), Dulbecco's modified Eagle medium (DMEM), 0.25% trypsin, Neurobasal and B27 media were from Invitrogen. The monoclonal mouse anti-hemagglutinin (HA, clone 12CA5) was prepared at the microscopy service of the Centro de Biología Molecular ‘Severo Ochoa’ (CBMSO, Madrid, Spain). Rabbit anti-DAT, goat anti-M6a, PI(3,4)P_2_(1,2-dioctanoyl), PI(4,5)P_2_(1,2-dihexanoyl) and PI(3,4,5)P_3_(1,2-dioctanoyl) were purchased from Santa Cruz Biotechnology. Anti-PtdIns(3,4)P_2_ IgG was from Echelon Biosciences. Sheep anti-SHIP2 was from RD systems, and Alexa-labeled secondary antibodies and TurboFect transfection reagent were obtained from ThermoFisher Scientific (Waltham, MA). The pGEM-T easy cloning vector was purchased from Promega (Madison, WI). All other chemicals, including oligonucleotides, were obtained from Sigma. Wistar rats were bred at the CBMSO, and experiments were performed in accordance with the Spanish Royal Decree 1201/2005 for the protection of animals used in scientific research and the EU guidelines laid out in directive 2010/63/EU.

### Plasmid constructs

The BirA* and BirA*-DAT constructs in pCDNA5/FRT were prepared as previously described [50]. The following expression vectors were kindly donated: SHIP2 (C. Erneux, Université Libre de Bruxelles, Brussels, BE), EGFP-M6a and EGFP-M6b (S. Hirata, National Institute of Genetics, Mishima, JP), Flag-Cullin1 (Z.Q. Pan, The Mount Sinai School of Medicine, New York, USA); EGFP-FBXL2 (M. Pagano New York University School of Medicine, New York, USA), EGFP-PGRMC1 and EGFP-PGRMC2 (M. Wheling, University of Heidelberg, GER). Some of them were myc-tagged by PCR amplification and subcloned into pCDNA3.1/MycHisA. Human 4F2hc, FBXO3, FBXO7, FBXL18 and xCT were amplified by PCR, using a human brain cDNA library as a template and the primers indicated in the supplementary Table 1. Amplified bands were cloned into a T-vector (pGEM-T easy) and then sub-cloned into either pCDNA3.1/MycHisA or a modified pCDNA3 expression vector carrying a previously inserted HA-epitope or EGFP, using standard cloning techniques. The biosensor NES–EGFP–cPHx3 was obtained from Addgene (plasmid 116,855); the mCherry-DAT construct was prepared previously [50].

### Generation of stable cell lines and in vivo biotinylation of bait-proximal proteins

Stable HT22 neuroblastoma cell lines expressing BirA* or BirA*-DAT were generated as previously described [46]. This cell line was chosen, since the dopaminergic line SH-SY5Y was reluctant to generate stable cell lines carrying the BirA* constructs. Briefly, HT22 cells were first engineered to create the Flp-In parental cell line carrying the pFRT/lacZeo vector inserted in the genome, according to the manufacturer’s instructions (Life Technologies). These parental HT22 cells stably expressed the lacZ-Zeocin fusion gene, and they were maintained in DMEM supplemented with 10% heat-inactivated FCS, 200 U/ml penicillin/streptomycin, 2 mM L-glutamine, 20 mM HEPES, 0.1 mM non-essential amino acids, 1 mM sodium pyruvate, and 100 μg/ml Zeocin (Invitrogen, Carlsbad, CA, USA). To insert the aforementioned pCDNA5/FRT expression vectors encoding BirA* or BirA*-DAT at the FRT site, the plasmids were transfected into the Flp-In parental HT22 cells using neofectin, together with the pOG44 recombinase expression vector. Stable HT22 transfectants were selected in the presence of 0.2 mg/ml hygromycin, and the selection medium was replenished every 3–4 days until discrete foci of hygromycin-resistant cells were evident after 3 weeks. Once selected, the stable cell lines were incubated with 50 μM biotin for 16 h and lysed in 4% SDS before purifying the biotinylated proteins using streptavidin-coated magnetic beads (Genscript). The BioID technique was performed as described previously [48], and eluted peptides were analyzed by liquid chromatography tandem MS (in the Proteomics unit at Centro Nacional de Biotecnología, CSIC) and peptide-mass fingerprinting, considering up to two biotinylations per peptide.

### Primary cultures of the brain cortex

Primary brain cortex cultures from 18-day-old rat fetuses were established as described elsewhere [51], isolating the tissue in Hanks balanced salt solution (Invitrogen), and dissociating the cells with 0.25% trypsin and 4 mg/ml DNase. The cells were incubated for 4 h in plating buffer (DMEM containing 10% FCS and supplemented with 10 mM glucose, 10 mM sodium pyruvate, 0.5 mM glutamine, 0.05 mg/ml gentamicin, 0.01% streptomycin, 100 µU/ml penicillin G) and the buffer was then replaced by culture medium (Neurobasal/B27 50:1 by volume, containing 0.5 mM glutamine). For transport and immunofluorescence assays, the cells were plated on poly-L-lysine (13 μg/ml)-coated 24-well plates at a density of 40,000 cells/well. Cytosine arabinoside was not added to allow glial proliferation, and the cultures were used after 14–15 day in vitro (DIV).

### Cell growth and transfection

HEK293 and SH-SY5Y cells (American Type Culture Collection) were grown in high-glucose DMEM supplemented with 10% FCS at 37 °C in an atmosphere of 5% CO_2_. Transient expression in HEK293 cells was achieved using TurboFect Transfection Reagent, according to the manufacturers’ instructions. The cells were incubated for 48 h at 37 °C, and then analyzed biochemically by immunofluorescence and/or transport assays. Primary neurons were transfected with Lipofectamine 2000 reagent at 12–13 DIV, according to the manufacturers’ instructions.

### Quantification of PI(3,4)P_2_ levels

Acidic lipids were extracted from SH-SY5Y or HT22 cells as described [52] and PI(3,4)P_2_-quantification assay was performed in accordance to the manufacturer’s instruction protocol by measuring samples in triplicate.

### Immunoprecipitation

Transiently transfected cells expressing tagged forms of DAT and the different preys were solubilized in RIPA buffer (50 mM Tris–HCl [pH 7.5], 0,1% SDS, 1% Triton X100, 0.5% sodium deoxycholate, 150 mM NaCl, 1 mM EGTA, 2 mM EDTA, 0.1 mM DTT) and the solubilized material was centrifuged at 12,000×*g* for 20 min. The supernatant was incubated overnight at 4 °C with the desired antibodies (2 µg/ml) and subsequently, 40 µl of protein A-cross-linked or protein G-cross-linked agarose beads was added, incubating the mixture for 1 h at 4 °C with constant rotation. The beads were washed five times with ice-cold lysis buffer before adding SDS–PAGE sample buffer to each sample. The bound proteins were dissociated from the beads by heating at 75 °C for 10 min before they were resolved on 10% SDS–PAGE gels as described below. HA or mCherry tags introduced in the N-terminus of DAT did not affect the immunoprecipitation output.

### Electrophoresis and immunoblotting

SDS–PAGE was performed on 7.5 or 10% polyacrylamide gels in the presence Laemmli buffer (65 mM Tris, 10% glycerol, 2.3% SDS, 100 mM DTT, 0.01% bromophenol blue). After electrophoresis, proteins were transferred to nitrocellulose membranes at 1.2 mA/cm^2^ for 2 h in a semi-dry electroblotting system (LKB) using a transfer buffer containing 192 mM glycine, 20% (v/v) methanol, 0,1% SDS and 25 mM, Tris–HCl (pH 8.3). Non-specific binding to the membrane was blocked by incubating the filter for 4 h at 25 °C with 5% non-fat milk protein in 10 mM Tris–HCl [pH 7.5], 150 mM NaCl and the membrane was then probed overnight at 4 °C with diluted primary antibody. After washing, antibody binding was detected with an IgG peroxidase-linked secondary antibody, and the labeled bands were visualized by ECL and quantified by densitometry on a GS-710 calibrated imaging densitometer from Bio-Rad using Quantity One software with film exposures.

### Cell surface biotinylation

Cells were plated at 70% confluence in 35 mm cell culture plates and transfected as indicated above. After 2 days, the cells were washed with ice-cold PBS (phosphate-buffered saline) and the cell surface proteins were labeled for 20 min at 4 °C by incubating them in a 1 ml solution containing the nonpermeable Sulfo-NHS–SS–Biotin reagent (1 mg/ml in PBS). The cells were then washed with 2 ml of PBS plus 100 mM lysine for 20 min to quench the reagent. After three additional washes with PBS, the cells were lysed for 30 min in 1 ml of RIPA buffer and the lysate was cleared by centrifugation at 14,000 g for 10 min. The biotinylated proteins were finally recovered by incubating the cleared lysate for 2 h at RT with streptavidin–agarose beads. After washing the beads three times with 1 ml of the lysis buffer, the protein bound to the beads was eluted in 2 × Laemmli sample buffer (10 min, 75 °C), separated by SDS–PAGE and analyzed in Western blotting.

### Transport assays

[^3^H]-DA transport in transfected cells was measured as described previously [50]. Briefly, the culture medium was replaced by complete phosphate buffered saline (PBS: 137 mM NaCl, 2 mM KCl, 8 mM Na_2_HPO_4_, 1.5 mM KH_2_PO_4_, 0.1 mM CaCl_2_, 1 mM MgCl_2_ and 10 mM glucose, 0.1 mM pargyline, 1 mM tropolone, 0.1 mM ascorbic acid, pH 7.4), and the cells were pre-incubated for 5 mins before addition of the radioactive medium containing 10 nM [^3^H]-DA (and variable amounts of DA for kinetics analyses) in complete PBS. Assays were stopped by aspiration followed by two washes with ice-cold PBS. Cells were lysed in 0.2 M NaOH, and accumulated radioactivity was measured in a liquid scintillation counter (1450 Microbeta Trilux, PerkinElmer). Finally, the background uptake values measured in mock-transfected cells were subtracted. Protein concentrations were determined using the Bio-Rad Protein Determination kit with bovine serum albumin (BSA) as the standard. The same procedure was used to determine [^3^H]-DA uptake in SH-SY5Y cells, except that background was corrected by subtracting uptake measured in a sodium-free PBS (NaCl iso-osmotically substituted by choline chloride). Each experiment was repeated at least three times. To determine uptake of [^3^H]-DA in synaptosomes, they were prepared from adult rat brain striatum as previously described [53]. Then, samples of synaptosomes were incubated with 100 nM [^3^H]-DA for 5 min. Uptake was terminated by the addition of 5 ml of cold Ringer's solution, and the synaptosomes were harvested onto nitrocellulose filter. After two additional washes, radioactivity that was collected on filters was counted with a liquid scintillation counter. Values were corrected by subtracting the background observed in the presence of 500 μM cocaine.

### Immunofluorescence

Primary cultures of neurons (transfected or not) or transfected cell lines were fixed in 4% paraformaldehyde/PBS, blocked, permeabilized, and incubated with primary and secondary antibodies as described previously [51]. The coverslips were mounted in fluoromount-G for analysis. Images were collected on an LSM510 confocal microscope coupled to an Axiovert200 M inverted microscope (Zeiss).

### In vivo imaging and image analysis

Primary cultures were transfected with expression vectors for mCherry-DAT and the PI(3,4)P_2_ biosensor NES–EGFP–cPHx3 [54]. Fluorescent neurons were imaged in 1.6 ml FluoroBrite DMEM (Life Technologies) supplemented with 25 mM HEPES (pH 7.4). Confocal microscopy was performed on a Nikon A1R + inverted microscope with an A1R resonant scan head and fiber-coupled four-line excitation (Ex) LU–NV laser combiner equipped with 405-, 445-, 488-, 561-, and 640-nm lines. Images in Nikon nd2 format were imported into the open access image analysis package Fiji [55]. Images obtained from both in vivo and fixed immunoreacted samples were analyzed for colocalization of fluorescent signals using various plugins available in this software. The ‘Plot Profile’ tool was used to analyze the distribution of fluorescence in neurites by drawing lines along the region of interest (ROI). Intensity values for each fluorescent protein were normalized by subtracting the minimum value along the ROI and dividing the data set by its maximum value. Pearson’s coefficients were calculated from ROIs of the acquired images using JACoP (Just Another Colocalization Plugin) with manual threshold. In addition, we used the plugin ‘Colocalization Colormap,’ which calculates the normalized mean deviation product (nMDP) as a measure of correlation between corresponding pairs of pixels according to Gorlewicz et al*.* [56], generating a visual map of colocalization.

### Electrophysiology

HT22 cells stably expressing BirA*-DAT were grown in 15-mm poly-L-lysine-coated coverslips. Whole-cell currents were recorded at room temperature (25 °C) in voltage-clamp configuration using a MultiClamp 700B amplifier (Molecular Devices). I–V curves were obtained in the absence and presence of DA (10 µM), calculating the substrate current (*I*_DA_) as the difference between the current in the presence of the substrate and the current in the absence of substrate (basal). Cells were perfused with extracellular solution containing 140 mM NaCl, 2.4 mM KCl, 2 mM CaCl_2_, 1 mM MgCl_2_, 10 mM glucose, 1 mM sodium L-ascorbate and 10 mM HEPES (pH 7.4). Patch recording pipettes (4–6 MΩ) were filled with a solution containing 155 mM potassium gluconate, 4 mM KCl, 5 mM MgATP, 0.1 mM EGTA and 10 mM HEPES (pH 7.4). Data were sampled at 20 kHz with a Digidata 1440A A/D converter (Axon Instruments) and filtered at 4 kHz. HT22 cells were voltage clamped at – 50 mV, and cells with series resistance > 20 MΩ or with more than 20% changes in series resistance throughout the experiment were discarded. All data were recorded and analyzed with pCLAMP 10 software (molecular devices).

## Results

### Identification of potential interactors of DAT

In previous works, we described the application of a proximity labeling technique, termed BioID, to uncover potential interactors of neurotransmitter transporters for glutamate and DA (GLT-1 and DAT, respectively). Thus, from the parental neural cell line HT22 we developed several stable lines expressing these transporters fused to the biotin ligase BirA*, as well as a control cell line expressing BirA* alone [49]. In these cell lines, the addition of biotin promoted biotinylation of a number of proteins that permanently or transiently resided in the neighborhood of the bait. These biotinylated proteins were isolated with streptavidin agarose beads and, after exhaustive washing, the beads underwent trypsin digestion and peptides in the mix were characterized by LC/MS. Proteins interacting with BirA*-GLT-1 and common interactors for BirA*-GLT-1 and BirA*-DAT have been characterized elsewhere [49, 50]. Peptides that were exclusively found in the BirA*-DAT samples are listed in Table 1, and some of them are analyzed in this article. We focused our attention on proteins that yielded at least two distinct peptides or a single biotinylated one in the LC/MS assay. The reduced list includes three integral plasma membrane proteins: the 4F2 cell-surface antigen heavy chain (Slc3a2), the membrane-associated progesterone receptor component (PGRMC2) and the neuronal membrane glycoprotein M6a (also termed Gpm6a). The list also contains a cytoplasmic component of the ubiquitination machinery termed F-box/LRR-repeat protein 2 (FBXL2) and the phosphatase phosphatidylinositol 3,4,5-trisphosphate 5-phosphatase 2 (Inppl1/SHIP2).

**Table 1:** Proteins identified by BioID and their primary localization

Accession code	Protein name	Peptide sequence	Modifications	Main localization
Q80UU9	Membrane-associated progesterone receptor component	GLCSGPGAGEESPAATLPR ILLAVNGK DFSLEQLR		ER and nucleus
P10852	4F2 cell-surface antigen heavy chain	VAEDETEAGVK IGDLQAFVGR		Membrane
P35802	Neuronal membrane glycoprotein M6-a	QFGIVTIGEEKK DLYGDFK		Membrane
Q6P549	Phosphatidylinositol 3,4,5-trisphosphate 5-phosphatase 2	PLSFPPPR EAFCQLLQLMK		Membrane/cytosol
Q8BH16	F-box/LRR-repeat protein 2	EAFCQLLQLMK	Biotin(K)	Membrane geranylated
Q4U2R1	E3 ubiquitin–protein ligase HERC2	IPGAEGLR		Nucleus/cytoskeleton
Q8VHN7	G-protein coupled receptor 98	SLSLSLAR		Inhibitor adelylate cyclase
P04441	H-2 class II histocompatibility antigen gamma chain	KPTEAPPK		Membrane
P01631	Ig kappa chain V–II region 26–10	FSGSGSGTDFTLK		Secreted
O88844	Isocitrate dehydrogenase [NADP] cytoplasmic	LVTGWVK		Cytosol
Q3U9G9	Lamin-B receptor	LGAFDLK		ER and nuclear membrane
Q810U3	Neurofascin	NLILAPGEDGRLVCR		Membrane single pass
Q8BHN0	Protein phosphatase 1L	VQGILAMSR	ox (M)	Membrane single pass
Q3TLH4	Protein PRRC2C	ELEQQREK		Stress granule
Q8BL43	Ras association domain-containing protein 10	VHLDRMRR		Cytoskeleton/cytosol
P97379	Ras GTPase-activating protein-binding protein 2	ETRGGGDDR		Cytosol/stress granules
Q91YJ3	Thymocyte nuclear protein 1	NLSNYWLMKSEPESR	ox (M)	Nucleus
P15533	Tripartite motif-containing protein 30A	TFQAPDLK		Nucleus
Q8BW70	Ubiquitin carboxyl-terminal hydrolase 38	ELMDAITKDNK		Cytosol/nucleus
Q8BWR4	Ubiquitin carboxyl-terminal hydrolase 40	GWTAGSLR		Cytosol/nucleus
Q5DU14	Unconventional myosin-XVI	HQAPGTLSVQWAR		Cytosol

### Confirmatory studies by immunoprecipitation and colocalization assays

#### 4F2hc

**Fig. 1:**
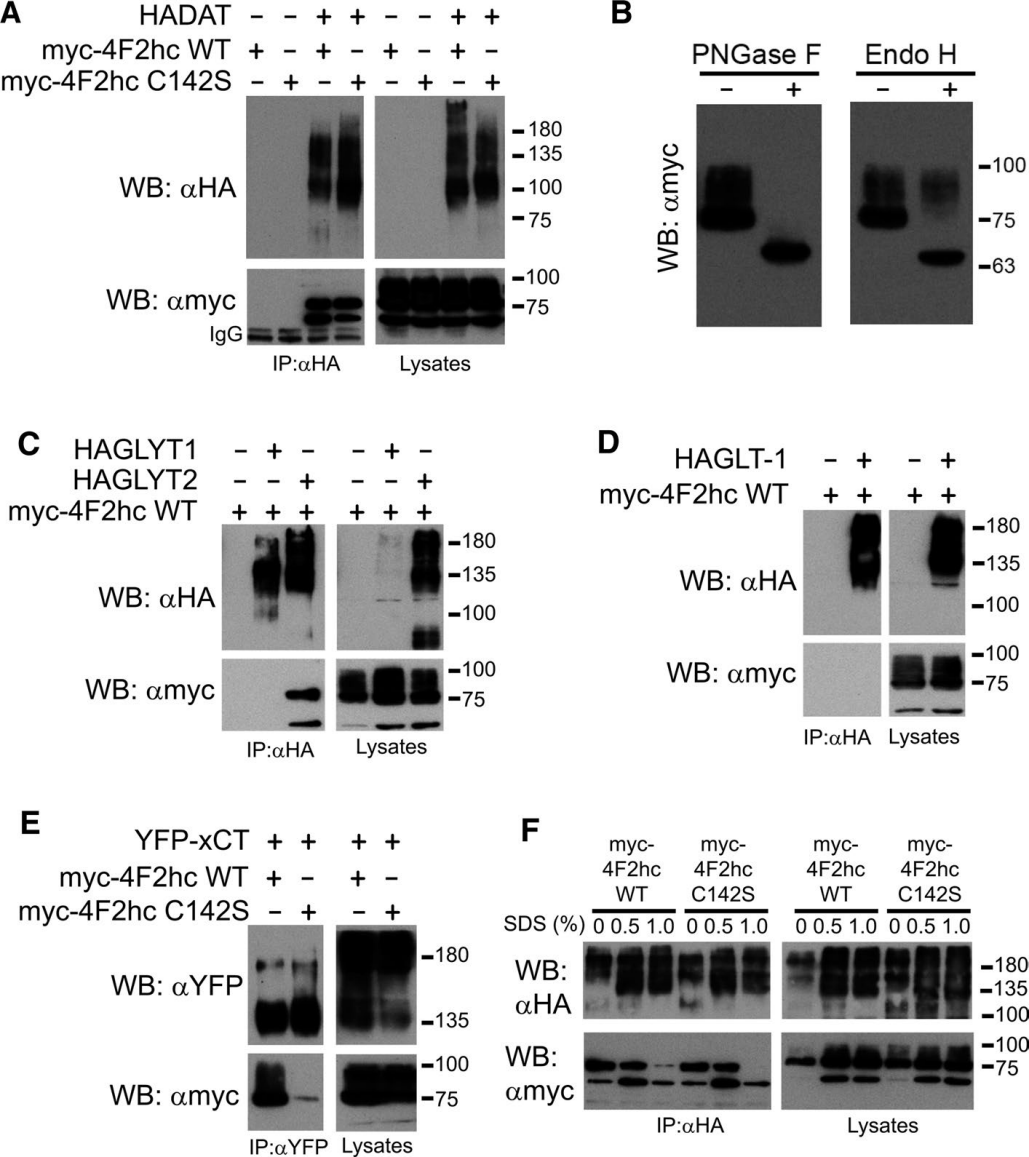
Interaction of the transmembrane chaperone 4F2hc with DAT and other transporters. HEK293 cells were transfected with either HA-DAT (**a,** **f**), HAGLYT1 (**c**), HAGLYT2 (**c**), HA-GLT-1 (**d**) or YFP-xCT (**e**) plus myc-4F2hcWT (**a, c, d, e**) or myc-4F2hcC142S (**a, e, f**) in the plasmid combinations indicated in the different panels. 48 h later, cells were lysed and extracts were immunoprecipitated with anti-HA (IP: αHA) or anti-YFP (IP: αYFP) antibodies, as indicated in the panels. Both the IP material and samples of the lysates were analyzed by western blotting. Immunoblots were probed with anti-HA (WB: αHA), anti-myc (WB: αmyc) or anti-YFP (WB: αYFP) to visualize the different tagged proteins. In **f**, cells were lysed with modified RIPA buffer (either without SDS or containing the indicated amount of SDS, in %). Then, lysates were diluted to reach a 0.1% SDS concentration and immunoprecipitated with αHA, probed with αmyc and re-probed with αHA. **b** Sensitivity of 4F2hc forms to the deglycosylating enzymes Endo H and PNGaseF was analyzed in extracts of HEK293 cells transfected with myc-4F2hc. Immunoblots were analyzed with anti-myc antibodies

4F2hc is a single-pass transmembrane protein with chaperone activity that represents the heavy chain of some heteromeric amino acid transporters. To test whether DAT might represent a novel partner of this chaperone, we first performed immunoprecipitation (IP) assays. HA-DAT and myc-4F2hc were transfected into HEK293 cells. We also included in the assay the mutant form myc-4F2hc C142S, since interaction of this chaperone with the light chains of heteromeric transporters is usually dependent on a disulfide bridge formed by this cysteine (C142 is equivalent to C91 of the short isoform used by the group of Palacín [57]) and an extracellular cysteine of the partner transporter. Then, the lysates underwent IP with anti-HA antiserum (Fig. 1a). The IP material produced two bands (65 and 75 kDa) for both myc-4F2hc WT and myc-4F2hc C142S whenever HA-DAT was present, indicating the existence of a bona fide interaction, but not mediated by a disulfide bridge. It must be noted that while the bands of the lysate lanes had a smeared appearance (spread from 75 up to about 100 kDa), only the 75- and 65-kDa bands were apparent in the IP lanes. These different bands (65 kDa, 75 kDa and the smears) corresponded to different glycosylation forms of myc-4F2hc, since treatment of the lysate with Endo H (which cleaves mannose-rich oligosaccharides from glycoproteins) decreased the size from 75 to 65 kDa (Fig. 1b). The smeared band above 75 kDa remained after the Endo H treatment, indicating that it corresponded to the completely mature form of myc-4F2hc carrying complex sugars added or modified in the cis and medial cisternae of the Golgi complex. Indeed, PNGase reduced the size of the 75–100 kDa bands to 65 kDa, (Fig. 1b), supporting that this band corresponded to immature myc-4F2hc located in the ER before its glycosylation. To appreciate specificity in the interaction between DAT and 4F2hc, we co-transfected the chaperone with other transporters, followed by IP with antibodies for the transporter. HA-GLYT2 (member of the SLC6 family) pulled-down the 75- and 65-kDa bands of myc-4F2hc. However, neither HA-GLYT1 (also belonging to SLC6 family) (Fig. 1c) nor HA-GLT-1 (SLC1 family) interacted with myc-4F2hc (Fig. 1d). Confirming previous reports [58], xCT (SLC7A11, GFP-tagged) pulled-down myc-4F2hc (Fig. 1e). However, unlike DAT, GFP-xCT interacted with all the glycosylation forms of myc-4F2hc, and this interaction was dependent on the presence of C142 (only a tiny band of 65 kDa remained in the IP material obtained with this mutant) (Fig. 1e).

To test whether the association between DAT and 4F2hc was dependent on hydrophobic interactions, we performed IP of the co-expressed proteins after solubilization in a modified RIPA buffer (either in the absence or presence of increasing concentrations of SDS). Solubilized material underwent IP (after dilution of SDS to 0.1% to avoid deleterious effect of SDS on the IP antibody) followed by western blot. Results indicated that while the 75-kDa band of myc-4F2hc was largely decreased in the immunoprecipitate by solubilizing with 1% SDS, the interaction of the 65-kDa one was resistant to the detergent (Fig. 1f). This suggest that the reticular form (65 kDa) is more intimately associated with DAT than the 75-kDa form or the fully glycosylated 4F2hc.

**Fig. 2:**
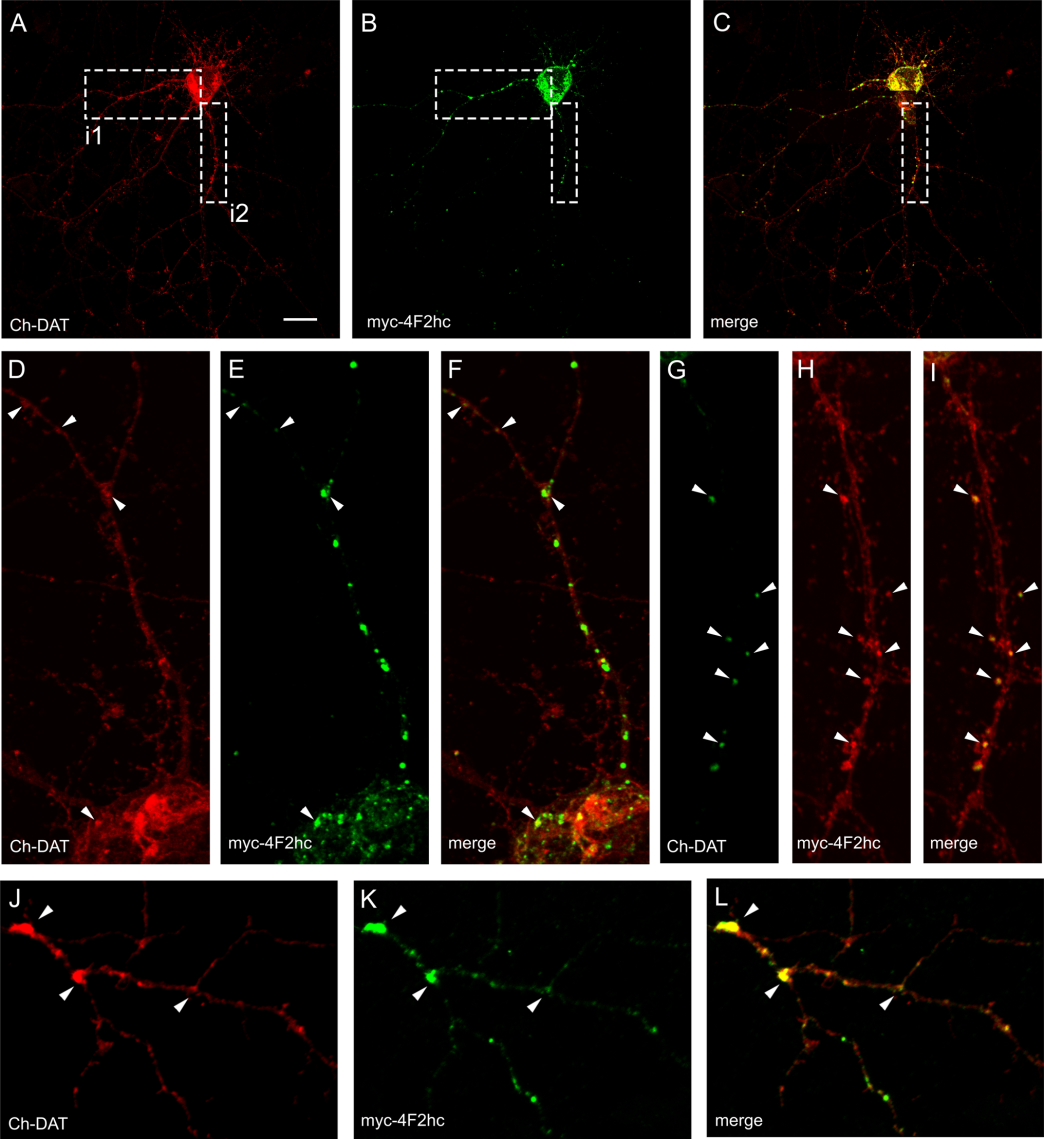
Localization of DAT and 4F2hc in neurons. **a–c** Primary cultures of cortical neurons were co-transfected at 13 DIV with mCherry-DAT and myc-4F2hc. 48 h later, cells were fixed and incubated with αmCherry and αmyc primary antibodies followed by Alexa-labeled secondary antibodies. Images collected by confocal microscopy correspond to distribution of mCherry-DAT (**a**, red channel), myc-4F2hc (**b**, green channel) or the merged one (**c**). **d–j** correspond to magnified insets i1 (**d–f**) and i2 (**g–i**). Colocalization of some puncta is highlighted by arrowheads. **j–l** correspond to another neuron similarly immunoreacted and arrowheads indicate clusters of both proteins located in ramification points of dendrites. Scale bar: 20 μm in **a–c**; 5 μm in **d–f** and **j–l**; 4 μm in **h–i**

Further indication of a close physical proximity between DAT and 4F2hc was seen in analysis of the immunofluorescence patterns in HEK293 cells and primary neurons transfected with mCherry-DAT and myc-4F2hc. Some co-transfected HEK293s showed intracellular vesicles containing both proteins (Fig. S1). Similarly, in primary neurons, we observed numerous intracellular clusters containing both proteins (Fig. 2). These clusters were distributed along the dendritic trees of neurons, consistent with an interaction of these proteins along the secretory pathway (Fig. 2d–f). Smaller clusters containing 4F2hc and DAT immunoreactivity were also observed in dendritic spines (Fig. 2g–i), and clusters containing both proteins were frequently observed at the branching points of dendrites Fig. 2j–l). Therefore, considering the subcellular localization and the IP assays, the results indicate that 4F2hc may act as a chaperone for DAT along the secretory pathway.

#### M6a

**Fig. 3:**
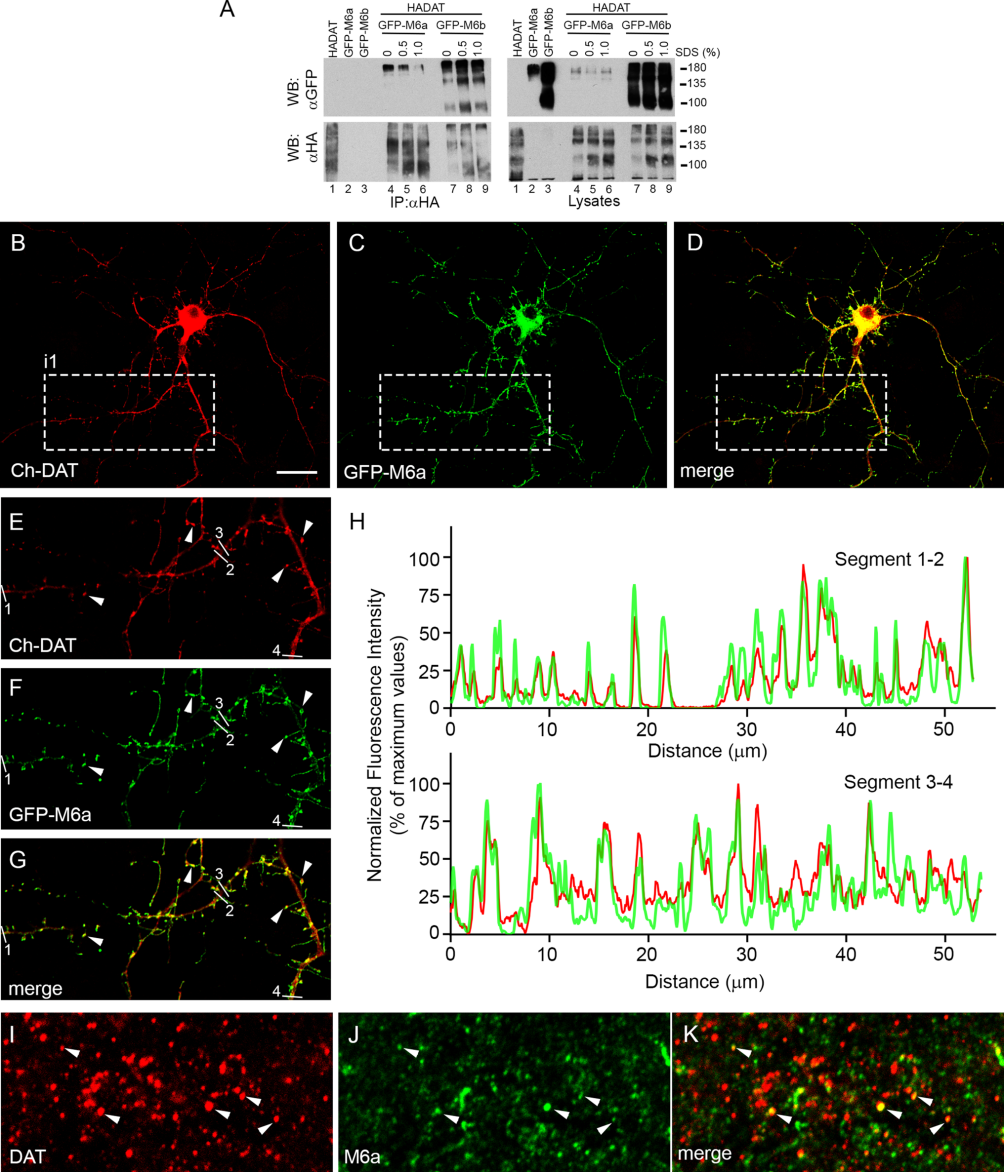
Interaction of proteolipid proteins M6a and M6b with DAT. **a** HEK293 cells were transfected with HA-DAT plus GFP-M6a or GFPM6b as indicated in the panel. 48 h later cells were lysed with RIPA buffer or a modified RIPA buffer (either without SDS or containing the indicated amount of SDS, in %).. The extracts were immunoprecipitated with anti-HA (IP: αHA). Both the IP material (left panels) and samples of the lysates (right panels) were analyzed by western blotting. Immunoblots were probed with anti-GFP (WB: αGFP) or anti-HA (WB: αHA) to visualize the tagged proteins. **b–g** Primary cultures of cortical neurons were co-transfected at 13 DIV with mCherry-DAT and GFP-M6a. 48 h later, cells were fixed and incubated with α-mCherry and α-GFP primary antibodies followed by Alexa-labeled secondary antibodies. Images collected by confocal microscopy correspond to distribution of mCherry-DAT (**b**, Ch-DAT, red channel), GFP-M6a (**c**, GFP-M6a, green channel) or the merged one (**d**). **e–g** Correspond to magnified inset i1. Colocalization of some puncta is highlighted by arrowheads. **h** Pixel fluorescence intensity along the linear region of interest 1–2 and 3–4 in **e–g** were calculated by manually drawing lines along these dendritic segments, and fluorescence intensity profiles were obtained from each individual color channel with the ‘Plot Profile’ tool of Fiji. Values were normalized to the maximum value in each segment after subtraction of the background (minimum values), and represented as percentages of maximum values. **i–k** Localization of DAT and M6a immunoreactivities in sections of rat striatum. Floating coronal sections were incubated with rabbit anti-DAT and goat anti-M6a, followed by the corresponding Alexa-labeled secondary antibodies. Images corresponding DAT (red channel) and M6a (green channel) were collected in a confocal microscope. Co-stained puncta, labeled by arrowheads, appear as yellow spots in the merged image. Scale bar: 20 μm in **b**–**d**; 10 μm in **e–g**; 4 μm in **i–k**

Another potential partner of DAT is the glycoprotein M6a, an integral membrane protein that is enriched in neuronal lipid rafts [59, 60]. M6a, like the closely related M6b, belongs to the tetraspan proteolipid protein family. IP of DAT from HEK293 cells co-transfected with HA-DAT and GFP-M6a revealed the existence of complexes containing both proteins (Fig. 3a). GFP-M6a was observed as a 180-kDa band, probably corresponding to trimeric association, since the Mr of the monomer is ~ 60 kDa. The amount of M6a in the complexes decreased after increasing the concentration of SDS in the solubilization solution (Fig. 3a, left upper panel, lanes 4–6). The efficiency of the IP of HA-DAT with anti-HA antiserum was confirmed by re-probing the blot with anti-HA (Fig. 3a, left lower panel). Control lane containing GFP-M6a but not HA-DAT (lane 2) revealed that as expected, the anti-HA antibody did not precipitate GFP-M6a. Control lysates confirmed the correct expression of HA-DAT and GFP-M6a in the expected lanes. Since it is known that the related proteolipid M6b interacts with the serotonin transporter [61], we also assayed the potential interaction of GFP-M6b with HA-DAT. Results indicate a pattern similar to that described for GFP-M6a, but the complexes were more resistant to increasing SDS concentration, suggesting a tight hydrophobic association (Fig. 3a, upper left panel, lanes 7–9).

Next, we investigated whether GFP-M6a colocalized with HA-DAT in transfected HEK293 cells and primary neurons. Consistent with data published for other cell types, the expression of M6a in HEK293 cells produced a large number of filopodia, where this protein was concentrated, as shown in the stack of images presented in Fig. S2. mCherry-DAT was also abundantly expressed in these filopodial structures (Fig. S2a–c, open arrowheads in images no. 2). Similarly, mCherry-DAT and GFP-M6a reached high levels in the substrate adhesive protrusions (Fig. S2a–c, arrowheads in images no. 3). In addition, both proteins colocalized in numerous clusters along the plasma membrane, as evidenced in the stack of images, both in the XY projection (Fig. S2a–c; montage of images 1–15) and orthogonal view of the stack (Fig S2d, i1 and i2). Nevertheless, mCherry-DAT was more evenly distributed than M6a, which was highly concentrated in these clusters. In primary neurons co-transfected with GFP-M6a and mCherry-DAT, both proteins were distributed along dendrites and axons. GFP-M6a was especially concentrated in dendritic spines and/or filopodia, in many of which mCherry-DAT was also present (Fig. 3b–g). Analysis of the precise distribution of clusters along these dendrites revealed an extensive overlap of fluorescence peaks for both proteins using the ‘Plot Profile’ tool of Fiji software (Fig. 3h). A similar co-staining of neuronal protrusions was observed for M6b and mCherry-DAT in transfected neurons (Fig. S3). To investigate whether M6a and DAT colocalized in brain tissue, floating sections of rat brains were incubated with polyclonal anti-M6a and monoclonal anti-DAT. In the striatum, M6a produced a punctate pattern that mainly did not colocalize with the puncta that were immunoreactive for DAT, although a small fraction of these puncta were immunoreactive for both proteins (arrowheads in Fig. 3i–k).

Altogether, these results indicate a tight interaction between M6a and DAT in co-transfected HEK293 cells and primary neurons, although the association in rat brain sections was limited.

#### PGRMC2

**Fig. 4:**
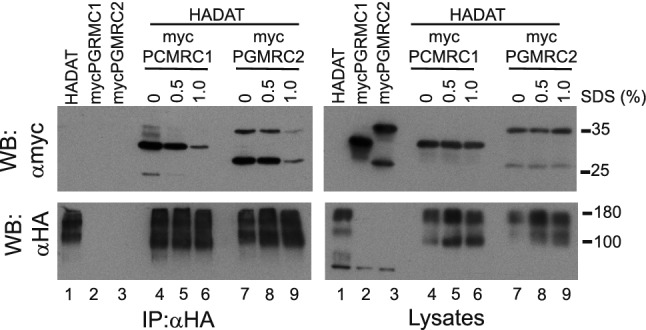
Interaction of membrane proteins PGRMC1 and PGRMC2 with DAT. HEK293 cells were transfected with pCDNA3 or HA-DAT plus myc-PGRMC1 or myc-PGRMC2 in the combinations indicated in the different panels. 48 h later, cells were lysed with a modified RIPA buffer (either without SDS or containing the indicated amount of SDS, in %), and the extracts were immunoprecipitated with anti-HA (IP: αHA). Both the IP material (left panels) and samples of the lysates (right panels) were analyzed by western blotting. Immunoblots were probed with anti-HA (WB: αGFP) or anti-myc (WB: αHA) to visualize the different tagged proteins

PGRMC2 and the related PGRMC1 are single-pass membrane proteins located in diverse membranous cellular structures (endoplasmic reticulum and plasma membrane) with other promiscuous interactions and pleiotropic (and uncertain) functions [62]. Again, we assayed whether DAT might be physically associated with these proteins after co-transfection in HEK293 cells followed by cell lysis and IP. Results indicated that both myc-PGRMC1 and myc-PGRMC2 were able to form immunoprecipitable complexes with HA-DAT when the transporter was pulled-down with anti-HA antibodies, although the interaction decreased with increasing SDS concentration in the solubilization solution (Fig. 4). Myc-PGRMC1 was expressed as a single band of 30 kDa, while myc-PGRMC2 displayed a double band of ~ 25 and 35 kDa. As expected, none of them was immunoprecipitated by the anti-HA antibody in the absence of HA-DAT (Fig. 4, control lanes 2 and 3). Control lysates confirmed expression of these proteins in the expected lanes, according to the composition of the transfection mixtures (Fig. 4, right panels). However, in marked contrast to the M6 proteins, we did not observe colocalization of these proteins with mCherry-DAT in transfected primary neurons, neither by visual inspection of the images nor using the ‘Plot Profile’ tool (except occasional puncta) (Fig. S4a–g).

#### FBXL2

**Fig. 5:**
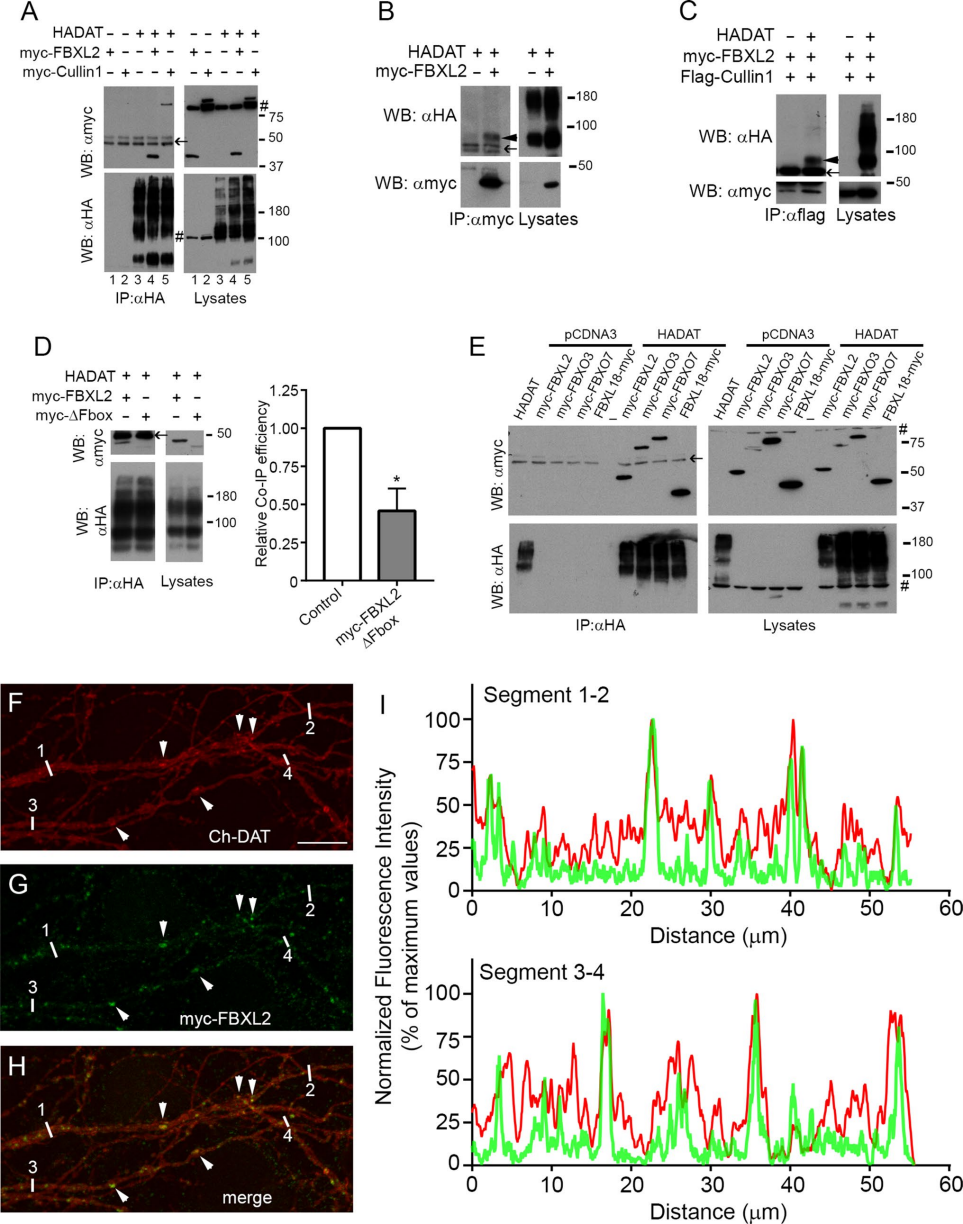
Interaction of F-box protein FBXL2 and components of the SCF complex with DAT. **a**–**c** HEK293 cells were transfected with HA-DAT, myc-FBXL2, myc-Cullin1 or flag-Cullin1 in the combinations indicated in the panels. 48 h later, cells were lysed and extracts were immunoprecipitated with anti-HA (IP: αHA in **a**), or anti-myc (IP: αmyc in **b**), or anti-flag (IP: αflag in **c**). Both the IP material (IP) and samples of the lysates (lysates) were analyzed by western blotting. Immunoblots were probed as indicated with anti-HA (WB: αHA) and anti-myc (WB: αmyc) to visualize the different tagged proteins. Arrowheads indicate the position of the immature HA-DAT band; # indicates unspecific bands occasionally appearing in the lysates and arrows indicate the position of the IgG bands. **d** Effect of the deletion of the N-terminus of myc-FBXL2 on the interaction with HA-DAT in HEK293 cells co-transfected and immunoprecipitated with the indicated constructs and antibodies, respectively. The co-IP efficiency (histograms) was determined by the ratio of the protein band intensities of immunoprecipitated myc-FBXL2 (WT or ΔFbox) to those of the corresponding lysates. The values were subsequently normalized with respect to that of myc-FBXL2 WT (control). Values are represented as the mean ± SEM of three independent experiments (*: paired *t* test, *p* < 0.05). **e** Study of the interaction of DAT with other F-box proteins. HEK293 cells were transfected with pCDNA3 or HADAT and the indicated myc-tagged F-box proteins. IP and western blotting was performed as indicated in **a–c**. **f–h** Immunofluorescence showing the localization of mCherry-DAT (Ch-DAT, red channel) and myc-FBXL2 (green channel) in neurons transfected with expression vectors for these constructs. Arrowheads indicate some colocalization spots. Note that the frame corresponds to the inset labeled in Fig. S5 as i1. Scale bar: 10 μm. **i** Pixel fluorescence intensities along the linear region 1–2 and 3–4 in **f–h** were calculated by manually drawing lines along these dendritic segments. Fluorescence intensity profiles were obtained from each individual color channel with the ‘Plot Profile’ tool of Fiji. Values were normalized to the maximum value in each segment after subtraction of the background (minimum values), and represented as percentages of maximum values

FBXL2, identified as a putative interactor in our BioID assay, is a member of the F-box protein family, which constitute one of the four subunits of the ubiquitin protein ligase complex called SCFs (SKP1–Cullin1–F-box). IP assays with anti-HA antibodies revealed that myc-FBXL2 was able to form stable complexes with HA-DAT (Fig. 5a, lane 4). Anti-HA also pulled-down myc-Cullin1 when the expression vector for this protein (and that of HA-DAT) was included in the transfection mixture (Fig. 5a, lane 5). However, FBXL2 and Cullin1 only interacted with the immature forms of the transporter. Thus, IP with anti-myc antiserum in extracts of cells expressing HA-DAT and myc-FBXL2 only pulled down a 60 kDa band of the transporter (Fig. 5b, arrowhead) and, similarly, anti-flag antiserum only immunoprecipitated this same band from extracts of cells expressing flag-Cullin1, myc-FBXL2 and HA-DAT (Fig. 5c, arrowhead).

The F-box domain is located in the N-terminus of this family of proteins, and it is normally used to interact with the SKP1-Cullin1 scaffold. To investigate whether it was the F-box of FBXL2 that mediated the interaction, we prepared a truncated mutant lacking this domain (Δ1-56). This truncation destabilized the protein, clearly decreasing its levels in cell lysates compared to the whole protein (Fig. 5d). We calculated the co-IP efficiency as the intensity ratio of the IP bands relative to the corresponding bands in the lysate samples. Although the mutant still interacted with HA-DAT, the efficiency was reduced by more than 50% (histograms in Fig. 5d). Therefore, in view of the importance of the F-box domain in the interaction with DAT, we studied the possible association of the transporter with other F-box proteins that have been implicated in Parkinson disease (such as FBXO7 or FBXO18) [63, 64] or in the stability of FBXL2 (such as FBXO3) [65]. In the presence of HA-DAT, the proteins myc-FBXO3, myc-FBXO7 and myc-FBX18 formed immunoprecipitable complexes using the anti-HA antibody, something that was not observed in controls performed in the absence of the transporter (Fig. 5e).

Immunofluorescence patterns in primary neurons transfected with mCherry-DAT and myc-FBXL2 were consistent with the existence of a close proximity of these two proteins in intracellular compartments, presumably corresponding to trafficking vesicles. Thus, myc-FBXL2 displayed a punctate pattern that was quite intense in the cell body and more dispersed in neurites (see low magnification of a cotransfected neuron in Fig. S5a–c, and the amplified inset in Fig. 5f–-h). Many of these FBXL2-positive puncta were also enriched in mCherry-DAT, as visualized with the ‘Plot Profile’ tool (Fig. 5i, corresponding to segments 1–2 and 3–4 of Fig. 5f–-h).

Altogether, IP and immunofluorescence assays are consistent with a role of FBXL2 and perhaps other F-box proteins in the association of DAT with the quality control mechanism based on the Cullin1 scaffold.

#### SHIP2

**Fig. 6:**
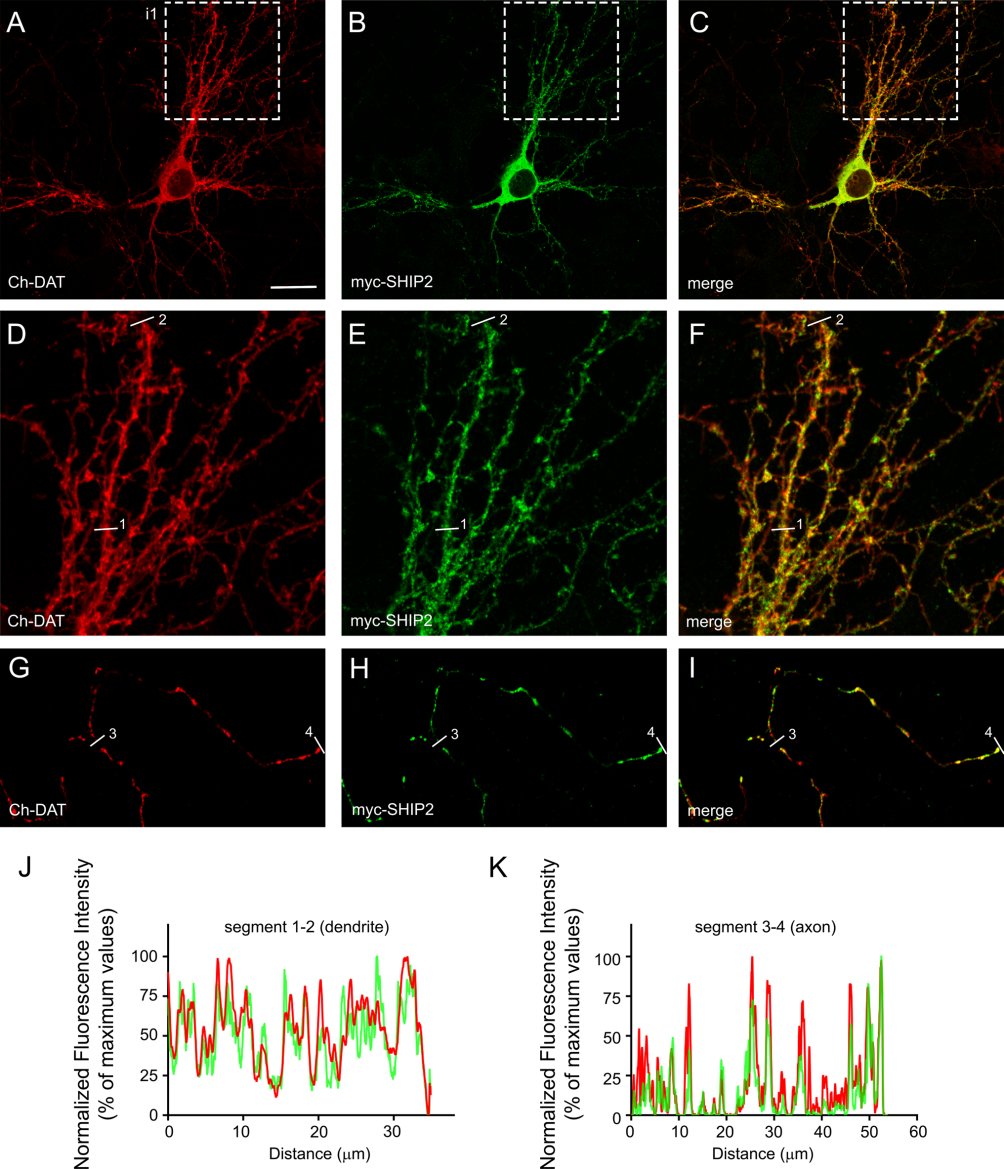
Colocalization of DAT and the phosphatase SHIP2 in transfected neurons. **a–c** Immunofluorescence showing colocalization of mCherry-DAT and myc-SHIP2 in neurons transfected with expression vectors for these constructs. **d–f** Correspond to a magnified view of the inset 1 (i1). **g–i** correspond to the axonal tract of a co-transfected neuron. **j–k** Pixel fluorescence intensity along the linear region 1–2 and 3–4 in **d–f** and **g–i** was calculated by manually drawing lines along these dendritic or axonal segments. Fluorescence intensity profiles were obtained from each individual color channel with the ‘Plot Profile’ tool of Fiji. Values were normalized to the maximum value in each segment after subtraction of the background (minimum values), and represented as percentages of maximum values. Scale bar: 20 μm in **a–c**; 8 μm in **d–i**

Another potential partner is SHIP2, a phosphatase that converts PI(3,4,5)P_3_ (phosphatidyl inositol 3,4,5 trisphosphate) into PI(3,4)P_2_. To confirm the existence of a physical or a functional interaction, first we tried to co-IP HA-DAT and myc-SHIP2 from lysates of HEK293 cells previously transfected with expression vectors for these proteins. However, under different IP conditions, results were negative (not shown). Nevertheless, immunohistochemical colocalization revealed that DAT and SHIP2 were closely located in specific subcellular structures, both in transfected neurons and in native tissue. Thus, visual inspection of images corresponding to neurons co-transfected with mCherry-DAT and myc-SHIP2 revealed the existence of an extensive co-clustering of both proteins along dendritic trees (Fig. 6a–c and the magnified inset in Fig. 6d–f) and along axonal tracts (Fig. 6g–i). This co-clustering was also observed by ‘Plot Profile’ analysis of dendrites and axons (Fig. 6j, k). Moreover, immunohistochemistry in rat brain slices revealed that both proteins were abundantly expressed in the striatum, although the expression of SHIP2 was broader than that of DAT (Fig. S6a–d). For instance, strong neuronal staining was observed for SHIP2 in the cortex (Fig. S6d), while DAT was barely detectable in that area of the brain (Fig. S6c). Higher magnification of the striatal region revealed that both antibodies stained axonal structures running through this region with abundant overlapping clusters (Fig. S6e–g). However, colocalization in punctate structures, presumably corresponding to terminals, was rare (Fig. S6h–j).

### Functional studies

**Fig. 7:**
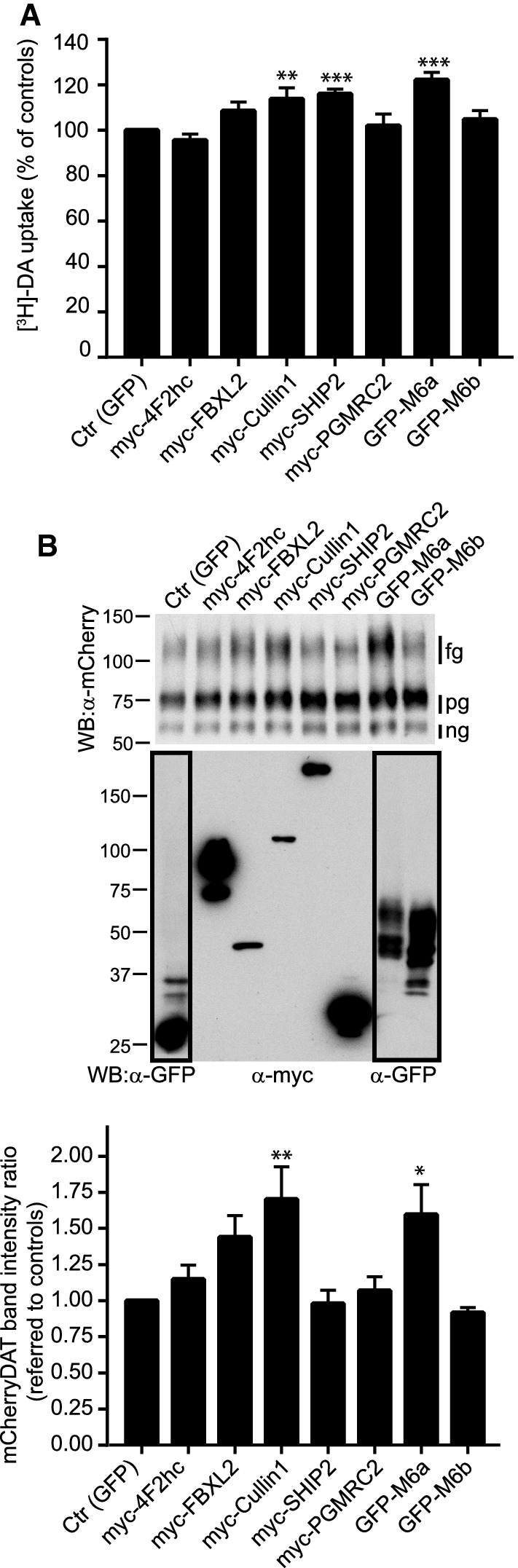
Functional effect of putative interactors. Dopamine uptake assay performed on HEK293 cells transiently transfected with expression vectors encoding mCherry-DAT and the expression vectors for the indicated constructs: myc-4F2hc, myc-FBXL2, myc-Cullin1, myc-SHIP2 myc-PGRMC2, GFP-M6a, GFP-M6b or pEGFP (the later in the controls). The ratio mCherryDAT:prey was 1:3. Transport was determined in the presence of 0.01 µM [^3^H]-DA for 10 min. Histograms represent the mean ± SEM from at least three independent experiments, each tested in triplicate. Values are expressed as percentage of the controls and were compared using one-way ANOVA with Dunnett’s multiple comparison test (**p < 0.01; ***p < 0.001). **b** Immunoblotting analysis of the effect of the different preys on the expression of mCherry-DAT. Cells were transfected as in **a**, and processed by immunoblotting with anti-mCherry (upper blot). Samples of the lysates were also analyzed for the expression of the preys with anti-GFP or anti-myc (lower blot). mCherry-DAT band intensities corresponding to fully glycosylated transporter (fg), or to partially glycosylated (pg) or to non-glycosylated (ng), were quantified with the Fiji software. Intensity ratios between fg and (pg + ng) bands were calculated and normalized against those determined in the controls. Values were represented as histograms that corresponded the means ± SEM from four independent blots and were compared using one-way ANOVA with Dunnett’s multiple comparison test (*p < 0.05; **p < 0.01)

Since most of the identified proteins seemed to have bona fide interactions (except SHIP2) or at least were physically located in the neighborhood of DAT, we investigated their effects on the uptake function of the transporter. To this end, HEK293 cells were co-transfected with mCherry-DAT and one of the following expression vectors: myc-4F2hc, GFP-M6a, GFP-M6b, myc-PGRMC2, myc-FBXL2, myc-Cullin1, myc-SHIP2 or pEGFP (the later, in the controls). 24 h after transfection, DA uptake was measured for 10 min at 37 °C. Of the tested constructs, GFP-M6a, myc-Cullin1 and myc-SHIP2 each produced a significant increase in the incorporated radioactivity, whereas myc-4F2hc, GFP-M6b and myc-PGRMC2 had no measurable effect (Fig. 7a). Myc-FBXL2 produced a slight increase that did not reach statistical significance. To clarify the reasons for these changes in DAT activity, we measured expression levels of mCherry-DAT in the presence of the different preys by western blot (Fig. 7b). Densitometric values of the fully glycosylated transporter (upper bands), presumably corresponding to mature protein located on the cell surface, where compared with intensity values of the partially glycosylated plus non-glycosylated intracellular protein (lower bands). The calculated ratios indicated that myc-FBXL2, myc-Cullin-1 and GFP-M6a increased levels of the fully glycosylated protein. These ratios were not affected by myc-SHIP2, myc-4F2hc or myc-PGMRC2. These results suggest that FBXL2, Cullin1 and M6a act on the activity of the transporter either by favoring the arrival of the transporter to the cell surface or by hindering its removal. However, SHIP2 appears to operate by a different mechanism, as it activates transport without any apparent change in the amount of mature protein.

**Fig. 8:**
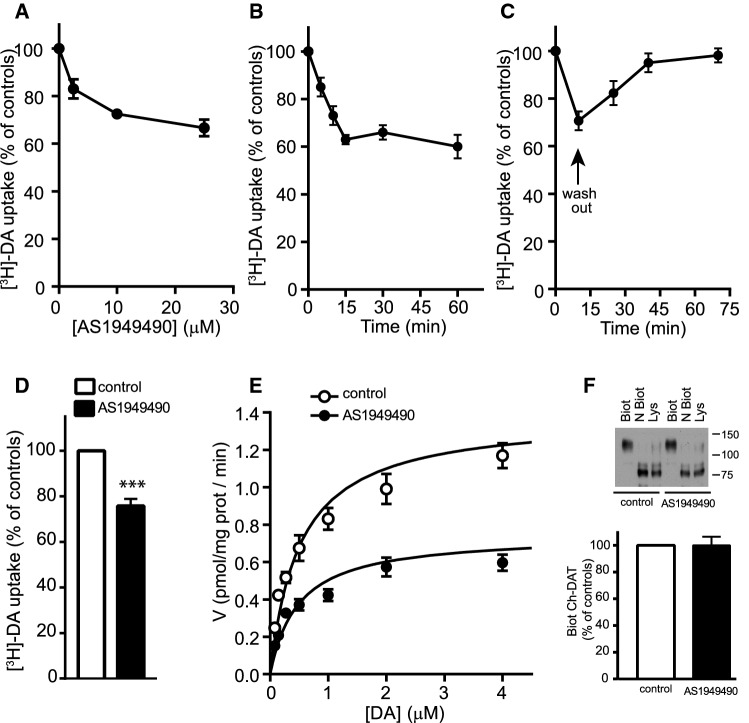
Effect of SHIP2 inhibition on DAT activity. **a** HT22 cells stably transfected with BirA*-DAT were preincubated for 30 min in the presence of the indicated concentrations of the SHIP2 inhibitor AS19494490. Transport was determined in the presence of 0.01 µM [^3^H]-DA for 10 min. Values correspond to the mean ± ESM of three triplicate determinations and are presented as a percentage of the untreated controls. **b** SH-SY5Y cells were incubated for the indicated times in the presence of AS1949490 (10 µM) and [^3^H]-DA; uptake was measured and data are presented as in **a**. **c** SH-SY5Y cells were incubated for 10 min in the presence of AS1949490 (10 µM) and then the compound was washed out for the indicated times; [^3^H]-DA transport was measured for 10 min as in **a**. **d** Synaptosomes isolated from rat striatum were preincubated for 10 min the presence of AS1949490 (10 µM) and then incubated in the presence of 0.01 µM [^3^H]-DA for 10 min (***p < 0.001, unpaired *t *test). **e** [^3^H]-DA uptake kinetics in SH-SY5Y cells in the absence (**○**) or presence of AS1949490 (10 µM, 20 min) (●). Values correspond to the mean ± SEM of two quadruplicate determinations. **f** HEK293 cells were transfected mCherry-DAT and 2 days later they were incubated in the absence or presence of AS1949490 (10 µM, 20 min). Cells were subjected to NHS–SS–biotinylation as described in Materials and Methods. Samples corresponding to biotinylated protein isolated with streptavidin–agarose beads (Biot), or non-biotinylated protein remaining after streptavidin–agarose precipitation (N Biot), or total protein in the lysates (Lys) were electrophoresed and immunoblotted,. Immunoblots were probed with anti-mCherry and the bands were visualized using the ECL method. Histograms represent the quantification of the intensities the biotinylated protein and are the average ± SEM of three experiments

Indeed, among the positive modulators, SHIP2 seems to be especially relevant, since this enzyme is involved in signaling cascades through modulation of phosphoinositide metabolism. Therefore, we decided to investigate in greater detail the observed functional interaction between DAT and SHIP2. To that end, we measured [^3^H]-DA uptake in HT22 cells stably transfected with BirA*-DAT and treated with AS1949490, a specific inhibitor of endogenous SHIP2. A 30-min pre-incubation of these cells in the presence of various concentrations of AS1949490 produced a significant reduction of uptake (Fig. 8a). A time-dependent inhibitory effect of AS1949490 was also observed in SH-SY5Y, a dopaminergic cell line that endogenously expresses DAT (Fig. 8b). Consistent with the reversible nature of the interaction between AS1949490 and SHIP2, the effect of the inhibitor was reversible. For instance, in SH-SY5Y cells, the 36% inhibitory effect produced by 15-min pre-incubation with the inhibitor disappeared after its removal by washing, and about 30 min later, the transporter had recovered to have an uptake activity similar to that observed in the untreated controls (Fig. 8c). The inhibitory effect of AS1949490 on [^3^H]-DA uptake was also observed in striatal rat brain synaptosomes (Fig. 8d). Kinetics analysis of DAT activity in SH-SY5Y cells revealed that AS1949490 reduced the *V*_max_ from 1.38 ± 0.06 pmol/min/mg prot in controls to 0.75 ± 0.04 pmol/min/mg prot in treated cells. However, the *K*_m_ was not significantly affected. The values were 0.51 ± 0.08 µM in control cells and 0.48 ± 0.09 µM in treated ones (Fig. 8e). To study whether the effect of AS1949490 was due to changes in the concentration of transporter on the cell surface, we carried out a biotinylation experiment of the protein present on that surface, using the nonpermeable reagent Sulfo-NHS–SS–Biotin. This experiment was carried out on HEK293 transfected with mCherry-DAT, which also respond to the inhibitor, since the expression level in SH-SY5Y is low to be able to determine it accurately with this technique. Results indicate that the inhibitory effect of AS1949490 was not due to a decrease in DAT levels on the cell surface (Fig. 8f). Altogether, these results indicate that inhibition of SHIP2 had an inhibitory effect on the kinetics of DAT, while overexpression of this phosphatase was stimulatory.

**Fig. 9:**
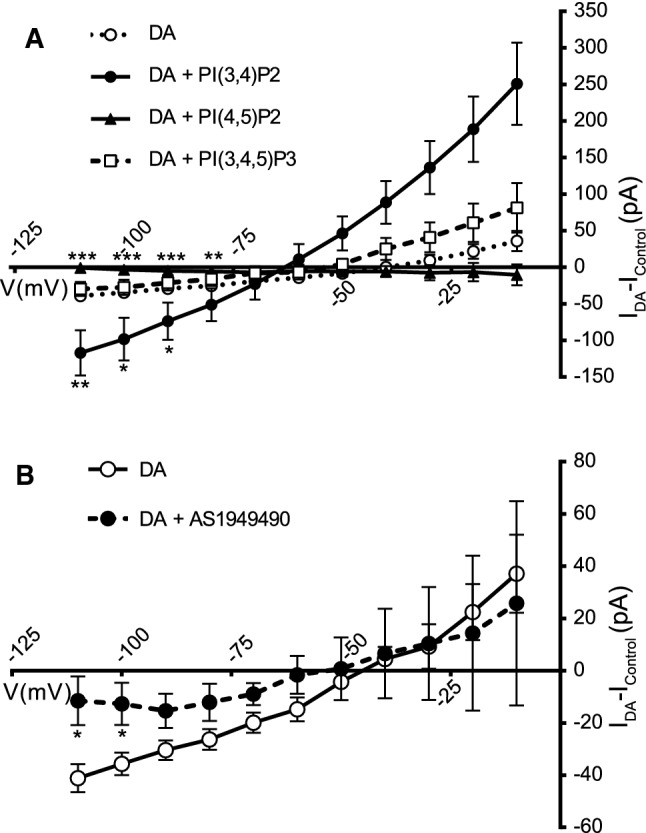
Effects of phophoinositides and a SHIP2 inhibitor on the currents associated with dopamine transport. **a** I–V curves were determined in the stable cell line HT22 BirA*-DAT in the whole-cell patch-clamp configuration. Currents were measured in the absence (**○**, *n* = 12) or the presence of 10 µM of PI(3,4)P_2_-diC8 (●, *n* = 8), or PI(4,5)P_2_-diC6 (▲, *n* = 9), or PI(3,4,5)P_3_-diC8 (□, *n* = 9) added to the recording solution. Values represent the difference of currents measured in the presence of dopamaine (DA; 10 µM) and those measured in its absence. Each data point represents the mean ± SEM of values obtained for the number of cells indicated for each condition. **b** Currents were measured as indicated in **a**; cells were preincubated for 10 min by perfusion of AS1949490 (10 µM) (●, *n* = 11) or vehicle (○, *n* = 9) into the recording chamber, and the treatments were maintained throughout the current recording. Currents were measured in the absence or the presence of DA (10 µM) and values were subtracted. Each data point represents the mean ± SEM of values obtained for the number of cells indicated for each condition. Statistical analysis in **a** and **b** was carried out using a two-tailed Student's *t* test for unpaired data (*p < 0.01; **p < 0.005; ***p < 0.001)

The inhibitory effect of AS1949490 might be attributed either to a decrease in the levels of PI(3,4)P_2_ or to an increase in PI(3,4,5)P_3_, or both. Therefore, to clarify the potential role of these two phosphoinositides, we assayed the effect of acutely administered water-soluble forms of these compounds on the electrophysiological currents induced by DA in HT22 cells stably transfected with BirA*-DAT. The DA substrate currents (*I*_DA_–*I*_Control_) were determined at different holding potentials (I–V curves) (Fig. 9a). The plot exhibited the typical behavior of DA transporter currents [66, 67], with the transport-associated depolarizing conductance increasing at more hyperpolarized potentials (i.e., for holding potentials more negative than − 50 mV) (Fig. 9a). The presence of PI(3,4)P_2_ potentiated DA-induced currents, whereas PI(3,4,5)P_3_ was ineffective. Notably, PI(4,5)P_2_, which is known to enhance amphetamine-induced efflux of DA, did not stimulate the Na^+^ currents, but rather had a weak inhibitory effect. Consistent with the radioactive uptake assays, there was a reduction in the currents determined in cells that had been pre-incubated with AS1949490 before the recordings (Fig. 9b). It is well established that inhibition of SHIP2 with this compound or with shRNA decreases the amount of PI(3,4)P_2_ in the MDA-MB-231 or SH-SY5Y cell lines [68, 69], and we also confirmed this effect of AS1949490 in the HT22 and SH-SY5Y cell lines (Fig. S8). Therefore, these electrophysiological data are consistent with a stimulatory effect of PI(3,4)P_2_ on DA uptake, making an inhibitory effect of PI(3,4,5)P_3_ unlikely.

**Fig. 10:**
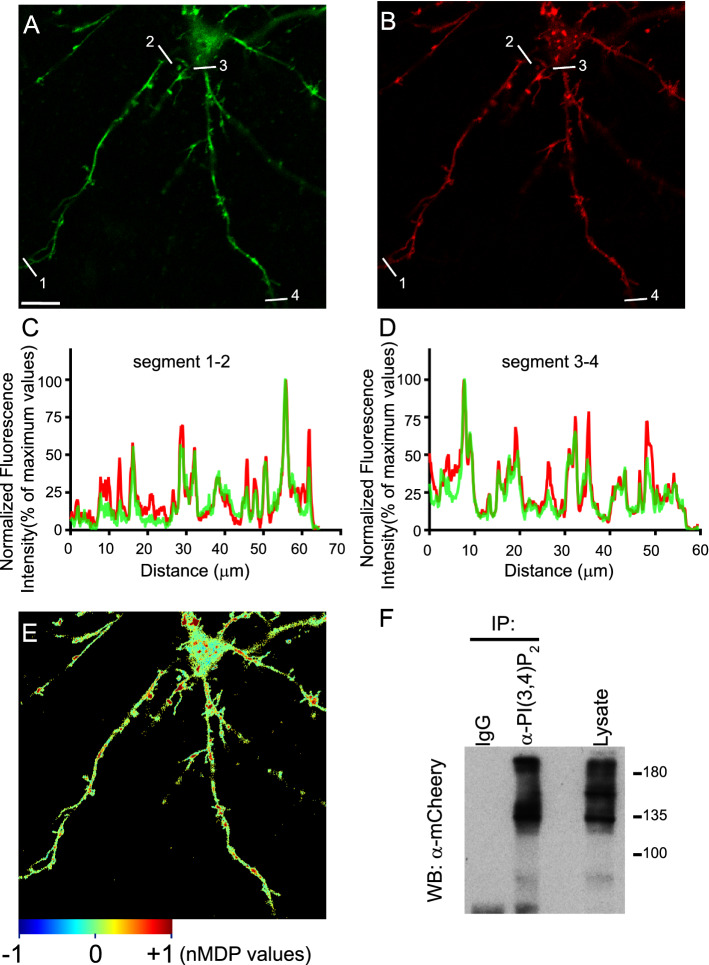
Study of the association of DAT and PI(3,4)P_2_. **a, b** Representative image obtained by live confocal microscopy showing the subcellular localization of DAT tagged with mCherry (Ch-DAT, red channel) and the PI(3,4)P_2_ biosensor NES–EGFP–cPHx3 (green channel). Primary neurons were transfected with expression vectors for both proteins at 12 DIV and 2 days later they were visualized by live-cell confocal microscopy. Scale bar = 10 μm. **c** Plot profile analysis of the regions of interest corresponding to segments 1–2 and 3–4 in **a**, **b**. **e** Colormap distribution of nMDP in the neuron presented in **a**, **b** showing colocalization in the hot color puncta. **f** HEK293 cells transfected with mCherry-DAT were solubilized and immunoprecipitated with anti-PI(3,4)P_2_ antibody or non-immune IgG. Immunoprecipitated material and a sample of the lysate were analyzed by western blotting with anti-mCherry (WB: αmCherry)

In addition, we found evidence for the existence of physical proximity between DAT and PI(3,4)P_2_ in living cells using a recently developed biosensor for this phospholipid, NES–EGFP–cPHx3 [54]. Cortical rat neurons were transfected with expression vectors for mCherry-DAT and NES–EGFP–cPHx3. Then, the transporter and the biosensor were visualized by live-cell confocal microscopy. In this way, we were able to locate numerous clusters located along dendrites and axons of transfected neurons, as evidenced by visual inspection of the images and with ‘Plot Profile’ analysis (Fig. 10a–d). This colocalization was not observed in neurons transfected with the biosensor and an unrelated mCherry-tagged protein (Fig. S7a-c). Moreover, quantitative analysis of images using the ‘JACoP’ plugin of Fiji software yielded a Pearson coefficient of 0.82 ± 0.02 (*n* = 20), consistent with an extensive colocalization of the transporter and the biosensor. A similar analysis of these images between mCherry-DAT and NES–EGFP–cPHx3 used the ‘Colocalization Colormap’ plugin of Fiji (see methods), based on a color scale in which negative values of the normalized mean deviation product values (nMDP) [56] are represented by cold colors (segregation) and values above 0 are represented by hot colors (colocalization). Numerous hot spot are visible along neurites (Fig. 10e). Furthermore, biochemical evidence for the association of PI(3,4)P_2_ and DAT was obtained by IP. Specific antibodies for this lipid, but not for non-immune IgGs, were able to immunoprecipitate mCherry-DAT from HEK293 cells transfected with the transporter (Fig. 10f).

Altogether, these data support the existence of a tight association of DAT and PI(3,4)P_2_ that results in a potentiation of transporter activity.

## Discussion

The dopamine transporter DAT plays an essential role in the control of dopamine activity and, therefore, in functions, such as control of movement, cognition, mood or reward. Likewise, DAT is the target of important drugs of abuse and pharmacological treatments. To a large extent, the pathophysiology of this transporter depends on its complex interactome. The aim of this work was to find new proteins that could interact with DAT. To that end, we have used a proximity labeling strategy in a neuronal line that stably expressed DAT fused to the ubiquitin ligase BirA*. In this way, we have identified three membrane proteins and two cytoplasmic proteins as potential partners of DAT: 4F2hc, M6a and PGRMC2, FBXL2 and SHIP2. Two of them, M6a and SHIP2, potentiated DA transport upon coexpression with DAT, as did the ubiquitinating system based in the Cullin-1 scaffold, which might be recruited to the transporter through FBXL2 or other F-box-related adaptors. FBXL2, Cullin1 and M6a also increased the amount of mature transporter, presumably located in the cell surface, suggesting that either they favor trafficking to the membrane or interfere with the removal of the transporter. The functional effects of 4F2hc and PGRMC2 remain uncertain. Paradoxically, the strongest effect was observed for SHIP2, the only one of the five proteins that does not appear to interact via direct protein–protein interaction, but rather through modification of the lipid environment of the transporter. Thus, SHIP2 and DAT did not co-IP under the various conditions tested nor affected the amount of transporter in the cell surface. However, this enzyme, which converts PI(3,4,5)P_3_ into PI(3,4)P_2_, showed a physical proximity to DAT both in cultured neurons transfected with DAT and SHIP2 and in brain tissue, where we observed numerous clusters of both proteins in axons that run through the striatum. In transfected neurons, DAT had a strong tendency to colocalize with the PI(3,4)P_2_ biosensor, NES–EGFP–cPHx3. This transporter–phospholipid association was robust enough to remain during phospholipid IP. On the other hand, functional tests revealed that PI(3,4)P_2_ depletion by pharmacological inhibition of SHIP2 led to a rapid decrease in transporter activity in different experimental systems including SH-SY5Y cells, which express DAT in a endogenous manner, and in striatum-derived synaptosomes. On the contrary, the addition of a water-soluble version of PI(3,4)P_2_ strongly increased the activity of DAT, as measured by electrophysiological recordings. This effect was specific to this phosphoinositide, and neither PI(4,5)P_2_ nor PI(3,4,5)P_3_ were able to mimic it. In this sense, PI(3,4)P_2_ plays essential roles in neurons that are clearly differentiated from other phosphoinositides, and this may underlie the asymmetric distribution of DAT in neurons. It has been described that DAT has a strong tendency to concentrate in axons and in structures, such as filopodia or at the end of neurites of PC12 cells [44, 70]. PI(3,4)P_2_ also accumulates at the tips of the filopodia and in patches of the neuronal membrane, where neurites are formed [71, 72]. In fact, the accumulation of PI(3,4)P_2_ in these clusters is a necessary and sufficient condition for the initiation of a new neuritic protrusion, a signal that neither PI(4,5)P_2_ nor PI(3,4,5)P_3_ are capable of mimicking [72]. We observed that DAT is abundantly located in HEK293 filopodia, while in neurons, the transporter and the PI(3,4)P_2_ biosensor were found to localize extensively in dendritic protrusions that correspond to filopodia and dendritic spines. The coincidence of DAT and PI(3,4)P_2_ in these highly curved structures may favor transporter activity during episodes of synaptogenesis in which these neuronal protrusions are involved [73, 74].

Previous studies have shown that DAT is also capable of interacting with PI(4,5)P_2_, a more abundant relative of PI(3,4)P_2_. However, this interaction does not affect neurotransmitter uptake itself, but rather it increases the DAT-mediated DA efflux promoted by amphetamines. PI(4,5)P_2_ electrostatically interacts with various positively charged residues on the transporter and promotes amphetamine-induced N-terminus phosphorylation to promote transporter conformations that mediate DA efflux [17, 38, 75]. Whether these types of interactions also mediate the effect of PI(3,4)P_2_ remains to be established, but the fact that the functional effect is very different suggests that the regulatory mechanism must be intrinsically distinct.

Our findings regarding the regulation of DAT by PI(3,4)P_2_ extend the already known interactions of DAT with the lipid environment and with the microdomain structure of the membrane itself, which plays an important role in aspects, such as lateral mobility or endocytosis. These properties seem critical to the function of this transporter for clearing neurotransmitter from the synaptic cleft. On the other hand, the association of DAT with PI(3,4)P_2_ may put it under the control of signaling pathways that affect this phospholipid. Although the signaling functions of PI(3,4)P_2_ are less well characterized than those of other phosphoinositides, it is known to be involved in the insulin-activated cascade [76]. An interesting possibility would be that PI(3,4)P_2_ mediates the effects on DAT trafficking described in alterations in the signaling of insulin in relation to obesogenic diets, or in motivational tasks induced by diet [77].

Among the other potential partners of DAT, M6a might favor DAT activity equally through its influence on the microdomain structure of the membrane, although further functional studies are needed to confirm this hypothesis in native systems. M6a is a palmitoylated neuronal proteolipid with strong tendency to accumulate in cholesterol and sphingolipid-enriched microdomains (lipid rafts). Expression of M6a in neurons promotes the growth of neurites and filopodia, participating in synaptogenesis [78–80]. Moreover, M6a is involved in the organization of rafts and signaling from these structures [59]. Consistently, we have observed that the expression of M6a in neurons notably increases the number of neuronal protrusions and the concentration of DAT in these structures. Therefore, one possibility is that DAT accumulates both in rafts and in neuronal protrusions by interacting with M6a, which could act as a carrier or scaffold for the transporter. Thus, M6a might be part of the cohort of palmitoylated binding partners including syntaxin 1A, flotillin 1 and DA receptors which may influence the raft partitioning of the transporter, not to mention palmitoylation of DAT itself, which is known to increase its concentration on rafts as well as its activity and stability [81]. In addition, other enzymes that influence the lipid composition of the membrane, such as the phospholipase PLPP2 that accumulates in the tips of neurites, might participate in the organization of the diverse microdomains that accumulate DAT [82]. Nevertheless, the expression of M6a in the adult brain is mainly localized to the axons of glutamatergic neurons in the hippocampus and cortex [83]. Indeed, we detected a rather sparse colocalization of M6a and DAT in the striatum. Even so, M6a might be transferred from glutamatergic neurons to neighboring neurons though extracellular vesicles in the adult brain [84]. Moreover, during embryonic development, M6a has been shown to be involved in differentiation and migration of diverse lineages from human and mouse neural stem cells, including tyrosine hydroxylase-positive catecholaminergic neurons [85, 86] and, consequently, it may participate in the early definition of membrane microdomains and in the partitioning of DAT. As revealed in M6a KO mice, the function of this protein is partially redundant with that of M6b, which is expressed both in neurons and in glia [60, 87]. Our results indicate that M6b is indeed also able to interact with DAT (albeit without any apparent functional effect), and previous studies have indicated that this isoform also interacts with the serotonin transporter [61]. Therefore, the interaction of these proteins with monoaminergic transporters might be relevant in condition, such as schizophrenia, bipolar disorders and claustrophobia, which have been associated with polymorphisms in the M6a gene [88–90], or with the learning and behavioral problems developed in patients with a de novo duplication of the M6a gene [91].

Unlike the other membrane proteins identified in our screening, 4F2hc is a type 2 transmembrane protein with a single transmembrane domain and a bulky extracellular globular domain. It acts as a chaperone and essential partner for the cell surface trafficking of at least six neutral amino acid transporters, to which it binds through hydrophobic interactions and a disulfide bridge, constituting the large subunit of these heteromeric transporter complexes. All these transporters belong to the SLC7 family, but they share a sequence homology of only 43% [92]. However, they seem to have in common the LeuT fold, which is more widespread in nature than was initially thought. In this study, we observed that two neuronal members of the SLC6 family (DAT and GLYT2) also display a strong interaction with 4F2hc that is resistant to SDS solubilization. This interaction seems to be stronger in the reticular and early Golgi compartments, since it was observed only with partially or non-glycosylated forms of DAT. In fact, immunofluorescence data indicated extensive colocalization of DAT and 4F2hc in intracellular vesicles, especially located along dendrites, including potential Golgi outpost located at the dendritic branch points. Although this interaction was not mediated by a disulfide bridge, it should be noted that structural data indicates that the hydrophobic effect is also relevant for interactions between heavy and light subunits in heteromeric amino acid transporters. In these transporters, the transmembrane domain of 4F2hc makes extensive interactions with the TM4 of the light subunit [93, 94]. By analogy, our data suggest that the chaperone properties of 4F2hc might help in the folding of DAT and perhaps other SLC6 transporters (namely, GLYT2) in neurons during their transit through the ER, possibly accommodating or avoiding undesired interaction during their biogenesis of hydrophobic segments, such as TM4. The necessity of chaperones for transporters carrying LeuT folds is also evident for SLC6A19, a neutral amino acid transporter expressed in the small intestine and kidneys that requires the chaperoning effect of ACE2. This interaction seems to be transient throughout the secretory pathway, and the proteins separate once on the cell surface [95]. However, we have been unable to find a functional effect on transporter activity, and additional studies are required to check whether the interaction between 4F2hc and DAT has a physiological significance. In fact, overexpression of 4F2hc can be counterproductive, since we have observed it is relatively toxic to neurons. The overexpressed protein accumulates in large intracellular dendritic clusters, where it may interfere with trafficking or interactions not only of transporters, but also of other proteins, such as integrins that are known to be partners of 4F2hc [96, 97].

Regarding FBXL2, it also formed immunoprecipitable complexes with immature forms of DAT in transfected HEK293 cells, and both proteins were colocalized in vesicular structures in co-transfected neurons, apart from the labelling obtained with BirA*-DAT. FBXL2 is a member of the F-box family of proteins, which are usually part of the SCF (SKP1–Cullin1–F-box) ubiquitination complex. One of the functions of the SCF complex is to eliminate misfolded proteins, and various components of the complex (SKP1, Cullin1, FBXL5, FBXO7, FBXL18, Parkin) have been associated with neurodegenerative diseases [63, 64, 98–100]. The formation of complexes containing DAT and several F-box proteins and Cullin1, together with the stimulatory effect of Cullin1 on transporter activity, suggests that this system may participate in quality control, eliminating misfolded DAT immature molecules and facilitating its intracellular trafficking. Indeed, this possibility would be compatible with the increased amounts of fully glycosylated transporter in the presence of both FBXL2 and Cullin1. Nevertheless, so far we cannot be sure that FBXL2 is the mediator for recruitment of Cullin1 to DAT, as other F-box proteins might also play this role. Addressing this question requires further research, considering the important function of the SCF complex and the recognized role of ubiquitination in the trafficking of DAT [36, 101, 102].

As a limitation of this study, we must mention that despite the fact that most of the preys identified by proximity labeling have been confirmed by immunoprecipitation in systems that overexpress the proteins of interest, the evidence that this occurs in native tissue is weak and requires more research. Proximity labeling not only marks direct interactors, but also weakly or transiently interacting proteins, which can make their detection difficult in native conditions, where protein concentrations are smaller.

In summary, in this article we have advanced the elucidation of the DAT interactome, an important protein in physiological processes, such as control of movement, cognition, mood and reward, as well as pathological conditions, such as Parkinson's disease, drug addiction and autism spectrum disorders. Not only did we discover new proteins with the potential to regulate the transporter's activity, but we also described a new regulatory function for the phospholipid PI(3,4)P_2_, which enhances the activity of DAT. The levels of this phospholipid are tightly regulated by hormones and signaling molecules, and may offer new drug targets for the treatment of the aforementioned dysfunctions.

### Supplementary Information

Below is the link to the electronic supplementary material.

Supplementary file1 (EPS 15329 KB)

Supplementary file2 (EPS 26404 KB)

Supplementary file3 (EPS 21044 KB)

Supplementary file4 (EPS 25010 KB)

Supplementary file5 (EPS 10980 KB)

Supplementary file6 (EPS 24869 KB)

Supplementary file7 (EPS 23580 KB)

Supplementary file8 (EPS 412 KB)

Supplementary file9 (DOCX 211 KB)

## Declarations

### Data availability

All data generated or analysed during this study are included in this published article [and its supplementary information files].
