# Supplementary material for: Identification by proximity labeling of novel lipidic and proteinaceous potential partners of the dopamine transporter
Source: Cell Mol Life Sci. 2021 Oct 28;78(23):7733–56. doi: 10.1007/s00018-021-03998-1 (PMC8629785; doi:10.1007/s00018-021-03998-1)
Supplement: Supplementary file 9 — Supplementary file9 (DOCX 211 KB) [file 18_2021_3998_MOESM9_ESM.docx]

**Supplementary figure legends**

**Fig. S1.** Localization of DAT and 4F2hc in transfected HEK293 cells. **a-c** HEK293 cells were co-transfected with mCherry-DAT and myc-4F2hc. 48 h later, cells were fixed and incubated with α-mCherry and α-myc primary antibodies followed by Alexa-labeled secondary antibodies. Images collected in a confocal microscope correspond to the distribution of mCherry-DAT (**a**, red channel), myc-4F2hc (**b**, green channel) or the merged image (**c**). Colocalization of some puncta is highlighted by arrows. Scale bar: 10 μm.

**Fig. S2.** Localization of DAT and M6a in transfected HEK293 cells. HEK293 cells were co-transfected with mCherry-DAT and GFP-M6a. 48 h later, cells were fixed and incubated with α-mCherry and α-GFP primary antibodies followed by Alexa-labeled secondary antibodies. **a-c** Montage of a stack of XY images collected in a confocal microscope, corresponding to the distribution of mCherry-DAT (red channel), GFP-M6a (green channel) or the merged image. Note the high staining observed in filopodia (open arrowheads) and in adhesive protrusions (closed arrowheads). **d** Insets i1 and i2 correspond to the reconstruction in the Z axis of the stack along the indicated Y and X axes (drawn only in images no. 2), respectively. Colocalization of some puncta is highlighted by arrows. Scale bar: 20 μm in **a-c**; 10 μm.in **d**.

**Fig. S3**. Localization of DAT and M6b in transfected neurons. Primary cultures of cortical neurons were co-transfected at 13 DIV with mCherry-DAT and GFP-M6b. 48 h later, cells were fixed and incubated with α-mCherry and α-GFP primary antibodies followed by Alexa-labeled secondary antibodies. **a** Merged images collected in a confocal microscope for mCherry-DAT (Ch-DAT, red channel) and GFP-M6b (green channel). **b-d** correspond to magnified inset i1. Colocalization of some puncta is highlighted by arrowheads. Scale bar: 20 μm in **a**; 8 μm in **b-d**.

**Fig. S4.** Localization of DAT and PGRMC2 in transfected neurons. Primary cultures of cortical neurons were co-transfected at 13 DIV with mCherry-DAT and myc-PGRMC2. 48 h later, cells were fixed and incubated with α-mCherry and α-myc primary antibodies followed by Alexa-labeled secondary antibodies. **a-c** Distribution of mCherry-DAT (Ch-DAT, red channel), myc-PGRMC2 (myc-PGMRC2, green channel) and merged image. **d-f** correspond to magnified inset i1. Colocalization puncta were rare (arrowhead). Scale bar: 25 μm in **a-c**; 12.5 μm in **d-f**. **g** Pixel fluorescence intensity along the linear region 1-2 indicated in **d-f** were calculated by manually drawing lines along the dendritic segment. Fluorescence intensity profile was obtained from each individual color channel with the ‘Plot Profile’ tool of Fiji. Values were normalized to the maximum value in the segment after subtraction of the background (minimum values), and represented as percentages of maximum values. Note that the arrowhead identifies the peak corresponding to the colocation point similarly marked in the **d-e** images.

**Fig. S5.** Localization of DAT and FBXL2 in transfected neurons. Immunofluorescence showing the localization of mCherry-DAT (Ch-DAT, red channel) and myc-FBXL2 (green channel) in neurons transfected with expression vectors for these constructs. Note that the inset is magnified in Fig. 5**f-h**. Scale bar: 20 μm.

**Fig. S6.** Localization of DAT and SHIP2 in the rat brain. Floating coronal sections of rat brain were incubated with rabbit anti-DAT and sheep anti-SHIP2, followed by the corresponding Alexa-labeled secondary antibodies. Confocal microscope images corresponding to DAT (**a, c, e, h**, red channel) and SHIP2 (**b, d f, i**, green channel) and merged image (**g, j**) were acquired in the striatum (**a-b**) or the cortex (**c-d**). **e-j** correspond to magnifications of the striatum. Note the existence of co-stained axonal structures (e-g, arrowheads), but its absence in puncta that presumably correspond to terminals (**h-j**). Scale bar: 500 μm in **a-d**; 5 μm in **e-g**; 4 μm in **h-j**.

**Fig. S7.** Distribution of the PI(3,4)P_2_ and an unrelated protein in transfected neurons. Primary neurons were transfected with expression vectors for the indicated proteins at 13 DIV and two days later, they were visualized. **a-b** Representative image obtained by live confocal microscopy showing the subcellular localization of the PI(3,4)P_2_ biosensor NES-EGFP-cPHx3 (green channel) and of mCherry-PGRMC2 (Ch-PGRMC2, red channel). Scale bar: 10 μm **c.** Plot profile analysis of the region of interest corresponding to the segment 1-2. Note the little overlap of both curves.

**Fig. S8.** Effect of SHIP2 inhibition on PI3,4)P_2_ levels. SH-SY5Y or BirA*-DAT HT22 cells were treated with AS1949490 (10 μM) or with vehicle (controls) for 20 min. Acidic lipids were extracted and quantifies as indicated in Materials and Methods. Histograms represent the mean ± SEM of three determinations. Statistical analysis was carried out using a two-tailed Student's t-test for paired data (*: p <0.01).

**Supplementary Table 1** Primer sequences

| Name | Primer sequence |
| --- | --- |
| EF2hc Fw | GGATCCATGAGCCAGGACACCGAGGTGGAT |
| EF2hc Rv | GCGGCCGCCCGGCCGCGTAGGGGAAGCGGAGC |
| FBXO3 Fw | GGTACCATGGCTGCCGTAGAGGCCGAGACGGGGCTGCTGACCCTG |
| FBXO3 Rv | GCGGCCGCAAACAGACGAGAGCAGCGGCGTCTGCG |
| FBXO7 Fw | GGTACCATGAGGCTGCGGGTGCGGCTTCTGAAG |
| FBXO7Rv | GCGGCCGCCATGAATGACAGCCGGCCATCAGTTG |
| FBXL18 Fw | GGATCCATGGCCAGCTCCGGAGAGGACATATCCAATGATGATG |
| FBXL18 Rv | TCAGCGACAGAGCAGCGAGTCTGGGGACAGGCAGTG |
| XCT Fw | GGTACCATGGTCAGAAAGCCTGTTGTGTCCAC |
| XCT Rv | TTAAGGATAATCAACCCGCGGTACTCTTTC |
| mycCull1 Fw | GGATCCATGTCGTCAACCCGGAGCCAG |
| mycCull1 Rv | GCGGCCGCTTAAGCCAAGTAACTGTAGGTG |
